# Supplementary material for: Comparative genomic analysis of six Glossina genomes, vectors of African trypanosomes
Source: Genome Biol. 2019 Sep 2;20:187. doi: 10.1186/s13059-019-1768-2 (PMC6721284; doi:10.1186/s13059-019-1768-2)
Supplement: Supplementary file 1 — Supplementary text and supplementary tables. (DOCX 186 kb) [file 13059_2019_1768_MOESM1_ESM.docx]

**Supplemental Text and Tables**

**Functional enrichment of *Glossina* specific genes (Figure 6 + Additional File 1 - Supplemental table 7)**

*Morsitans sub-Genus Specific and Universal Genes*

Analysis of genes specific and universal to the *Morsitans* sub-Genus (containing *G. morsitans*, *G. pallidipes* and *G. austeni*) revealed enrichment in proteasome activating ATPase function. In addition to the protease enrichment observed in the Genus at large, this may be an adaptation to aid in protein digestion. Proteasome function is also linked with the process of autophagy [104]. Autophagy plays an important role in the process of lactation in tsetse. The milk gland tissue of female flies undergoes autophagic renewal at the end of each gonotrophic cycle. This process breaks down and restores the secretory cells in the gland immediately after larval deposition to ensure optimal function during the next lactation cycle [105].

**Sub-genus specific gene family expansions and contractions (Figure 7, Additional File 1 - Supplemental Table 8 and Additional File 2 - Supplemental figures 10)**

*Palpalis sub-genus specific gene family variations*

A gene family encoding phosphoribosylformylglycinamidine synthases (VBGT00190000012418) is also increased in the *Palpalis* group. This enzyme, also known as *ade2* in *Drosophila*, is part of the *de novo* biosynthetic pathway for purine nucleotides. In tsetse, the function of this pathway is negatively impacted by the loss of the obligate *Wigglesworthia* symbiont and its associated B-vitamin cofactors, upon which nucleotide biosynthesis pathways rely [110, 111]. Purines in addition to being components of DNA act as essential enzymatic co-factors (ATP/GTP, S-adenosylmethionine). Amplification of an enzyme associated with *de novo* biosynthesis of purines reinforces the significance of their role in tsetse biology and may be an optimization of the pathway within the context of the symbiotic relationship with *Wigglesworthia*.

A family of thiamine (vitamin B1) dependent fatty acid alpha oxidases is also expanded within the *Palpalis* group (VBGT00190000011125). These enzymes function to remove single carbons from fatty acid chains in a thiamine dependent manner to facilitate beta-oxidation. They are also capable of acting on methylated fatty acids, which prevent beta-oxidation. Interestingly, the *Drosophila* ortholog to this gene is most highly expressed in the digestive tract of developing larvae, adult females and embryos. These enzymes could be important for larval milk digestion and are likely impacted by the thiamine deficiencies noted in *Wigglesworthia* free flies.

**Tsetse saliva genes (Figure 11)**

The analysis performed here encompasses five gene families encoding the following saliva protein classes: i) 5’nucleotidase-related proteins, ii) tsetse thrombin inhibitors, iii) tsetse salivary gland proteins, iv) adenosine-deaminases and v) the tsetse antigen-5 proteins. Comparison of these classes across these six tsetse fly species indicates that orthologues are present in all the genomes and that (in most cases) these sequences show sub-genus specific i.e. *Morsitans*, *Palpalis* and *Fusca* features. This agrees with previous findings documenting the differential sialome protein profiles of the respective sub-genera [127]. Sequences from *G. brevipalpis* show the strongest sequence divergence. This correlates with the *Fusca* group’s early evolutionary divergence in tsetse fly evolution.

In the host hemostatic reactions, ATP- and ADP-mediated platelet aggregation is essential and blood sucking insects have ATP/ADP-hydrolysing [ATP(D)ase; apyrase] enzymes present in their salivary secretions [128]. Each of the *Glossina* species examined here have three genes coding for proteins with a 5’-nucleotidase signature. These genes are located in a conserved 26 kb locus and include the *5’Nuc*, *sgp3* and *apyrase1-like* genes (Figure #A). A second *apyrase2-like* gene is located on a separate scaffold.

The 5’Nuc orthologues are highly conserved across the species with similarities ranging from 92%-94% within the *Morsitans* group, 97% within the Palpalis species. Similarity comparison of these sequences relative to *G. brevipalpis* revealed sequence similarity of ~70%. Analysis of the predicted translation products of the representative sequences from the *Morsitans* and *Palpalis* groups identified the first 25 amino acids of these proteins as a putative signal peptide corresponding to the secretory nature of saliva proteins [128]. However, no signal peptide was detected for the *5’Nuc* genomic sequence of the *Fusca* group flies. All the amino acids predicted in *G. morsitans* to be involved in catalytic activity, co-factor and substrate binding [128] are conserved amongst all tsetse species, with the exception of a substrate binding amino acid Arg^358^ that is replaced by phenylalanine in *G. austeni*. It is not clear whether the substitution will have an impact on its apyrase activity, immunogenic properties or vector competence.

**Neuropeptides and protein hormone receptors (Additional File 1 - Supplemental Tables 11+12)**

*Neuropeptides*

Neuropeptides are amino acid-based molecules that can enter into the circulatory system and act as signalling molecules as well as neuromodulators and neurotransmitters within the central nervous system. Neuropeptides regulate key biological processes including metabolism and homeostasis, reproduction, growth and development, behaviour and feeding amongst others. Previous work identified 39 neuropeptide genes in *G. morsitans* including the conserved core neuropeptide genes found in all insect genomes, as well as a variable cohort of neuropeptide genes only found in some insect genomes [136].

The current analysis expands the scope of the previous analysis of neuropeptides [137] and their receptors by including the new tsetse fly species and *M. domestica*. The housefly differs from tsetse across multiple aspects of their biology including reproduction, development and feeding biology. This provides the opportunity to determine if these differences are reflected in the neuropeptide/receptor complement.

The core set of neuropeptides are present in most of the species examined (Additional File 1 - Supplementary Table 11). *G. brevipalpis* is an obvious outsider in this analysis – consistently showing the lowest protein identity to *G. morsitans* and missing a total of 6 neuropeptide genes (Allatostatin B, CCHamide-1, DH (calcitonin), ETH, Leucokinin and PDF). These genes are present in the other *Glossina* species.

The lack of certain neuropeptides in *G. morsitans* is reinforced by the consistent absence in the other *Glossina* species. The missing peptide genes include adipokinetic hormone/corazonin-like neuropeptide (ACP - function unknown), allatotropin (stimulation of juvenile hormone synthesis and cardiac activity), inotocin (control of water balance) [138] and sulfakinin (control of feeding and of larval locomotion/odor preferences) [136]. Indeed, almost none of the above neuropeptides are associated with the genomes of the other *Glossina* species or in *M. domestica*.

This analysis also identified a number of neuropeptide genes not previously located or confirmed in the *G. morsitans* genome [23]. These include: Myoinhibiting peptide (allatostatin B) and CCH amide 1 (possible modulator of sensory perception and olfactory behavior in starved *D. melanogaster*) [139] which were located in the genome of *M. domestica* and all the *Glossina* species with the exception of *G. brevipalpis*. The orcokinin gene (regulation of circadian rhythms) is present in all the *Glossina* species but missing in *M. domestica*. In addition, some recently discovered neuropeptide families were included in the current gene searches, and so natalisin (regulation of reproductive behavior and fecundity) [140] and trissin (function unknown) are now documented in all six *Glossina* species.

*Neuropeptide Receptors*

Most neuropeptides carry out their function by activating specific G-protein coupled receptors (GPCRs), guanylate cyclase receptors and receptor tyrosine kinases [141]. We annotated over 40 GPCRs in the *Glossina* genome databases (Additional File 1 - Supplemental Table 12), including orphan receptors with as yet unknown ligand(s). We confirm here that the vasopressin/oxytocin related neuropeptide, inotocin, and its receptor were lost during dipteran evolution as this system is also absent in all other dipteran genomes investigated so far. The allatotropin, ACP neuropeptides and their receptors are also absent in the *Glossina* species, *M. domestica* and *Drosophila*, but occur in mosquito genomes, pointing to a more recent loss of these neuropeptidergic systems in the Schizophora clade. The sulfakinin receptor is missing in all the *Glossina* species suggesting evolutionary loss of this signaling system in tsetse flies possibly due to the evolution of tsetse’s blood feeding behaviour/biology.

**Cuticular Proteins (Additional File 1 - Supplemental Table 13)**

Query of the *Glossina* genomes with sequence motifs characteristic of cuticle protein families revealed 584 genes encoding putative cuticle proteins. Each species represented between 101-130 genes which were assigned to one of eight families (CPR, CPAP1, CPAP3, CPF, CPCFC, CPLCA, CPLCG, and TWDL; (Additional File 11). The total number of cuticle protein genes identified in *Glossina* is within the range observed in other insects (Supplemental table 13), but less than that of other dipterans [79, 142]. As with other insects, the number of CPR genes (60-85) constituted the largest group of cuticle protein genes in the *Glossina* genomes; however, this number is two- to fourfold less than in other dipterans and partially accounted for the reduced number of total cuticle protein genes. In general, the number of genes in each cuticle protein family did not correlate with evolutionary relatedness in the dipterans. *Glossina* had a similar number of genes in the CPAP1 and TWDL families as the mosquito species (Culicidae), whereas the number of CPLCG genes was more similar to the other Brachycera flies (Supplemental table 14). This variability in gene numbers is evident in the TWDL (Tweedle) family, which shows an expansion in the dipterans [79]. The TWDL genes tended to group by taxa within Diptera and gene expansions are evident in *Musca domestica*, *Drosophila melanogaster*, *Ceratitis capitata*; however, *Glossina* lack these large expansions. Further study is needed to determine if this difference is due to multiple, independent events of gene duplication within brachyceran flies, or if *Glossina* lost genes that were present in a common ancestor. Cuticular protein gene expansions likely reflects adaptive evolution [143]. The lack of expansion in *Glossina* may reflect their unique strategy of intrauterine larval development. The relative lack of cuticle protein synthesis during the first two larval instars of *G. morsitans* [144] further suggests that the protection of the mother limits the need for extensive cuticle proteins. However, there is currently a lack in understanding of the precise role of these protein families, and the functional implications of their reduction requires further elucidation.

**Transcription factors (Additional File 2 - Supplemental Figure 11)**

To compare repertoires of predicted DNA binding transcriptional regulatory proteins we searched the proteins from the tsetse genomes for putative DNA binding domains. These proteins were then classified according to the class of binding domain to provide a picture of the relative abundance of these important regulatory proteins. The analysis identified a total of between 700 (*G. morsitans*) and 806 (*G. fuscipes*) putative TFs in the *Glossina* genomes, which is similar to other insect genomes (e.g., 701 for *D. melanogaster*). Likewise, for the most part, the number of members of each *Glossina* TF family is comparable to that of other insects (Additional File 2 - Supplemental Figure 11).

We next inferred DNA binding motifs for these TFs using a previously described procedure [80]. For the following, we use *G. pallidipes*, which has the median number of TFs among the six species, as an example. Similar results were obtained for the other species. Of the 783 *G. pallidipes* TFs, we were able to infer motifs for 266 (34%) (Additional File 12), mostly based on DNA binding specificity data from *D. melanogaster* (215 TFs), but also from species as distant as mouse (3 TFs) and zebrafish (2 TFs). Many of the largest TF families have inferred motifs for a substantial proportion of their TFs, including Homeodomain (63 of 89, 71%), bHLH (47 of 52, 90%), and nuclear receptors (10 of 18, 56%). As expected, the largest gap is for C2H2 zinc fingers (only 32 of 270, ~12%), which evolve quickly by shuffling their many zinc finger arrays, resulting in largely dissimilar DBD sequences across organisms [145]. This catalog of TFs and their predicted DNA binding motifs offers an opportunity to begin examining and comparing gene regulatory networks in the *Glossina* genomes

**Peritrophic Matrix (Additional File 1 - Supplemental table 14)**

Peritrophins and peritrophin-like proteins (PLPs) [131, 132], typified by the presence of chitin-binding domains (CBDs), are important structural components of the peritrophic matrix (PM), a semi-permeable barrier separating the lumen from the midgut epithelial cells [133]. The insect PM is involved in regulating immunity genes and appear to act as a physical barrier to pathogens [134, 135].

Comparative analyses of peritrophins and PLPs across all six major *Glossina* species show a high degree of conservation with slight differences in the numbers and characteristics of these proteins. *Glossina* have less than half the number of peritrophins and PLPs relative to *Musca, Stomoxys* and *Drosophila*, with these latter Dipterans having similar numbers of genes to various mosquito species. The higher number of genes found in these species could reflect the major differences in the feeding habits between them and *Glossina*. The digestive tracts of *Glossina* are adapted to a restricted diet of lactation secretions and blood, while other Dipteran species have much more diverse diets and greater exposure to dietary pathogens and hazards. The streamlining of peritrophins in *Glossina* could potentially explain their susceptibility to infection by trypanosomes.

**Supplemental Tables**

**Supplemental Table 1:** *Glossina* strains and colonies of origin for sequenced *Glossina* species

| Species | Strain | Sample IDs | Location |
| --- | --- | --- | --- |
| *Glossina fuscipes* | IPCL* | MD7, MD7-2, MD8, MD8-1 | Institute of Zoology  Slovak Academy of Sciences |
| *Glossina austeni* | TTRI |  | Tsetse and Trypanosomosis Research Institute (TTRI) laboratories in Tanga, Tanzania |
| *Glossina pallidipes* | IPCL* | MD2 1-4 | Institute of Zoology  Slovak Academy of Sciences |
| *Glossina brevipalpis* | IPCL* |  | IPCL* laboratories in Seibersdorf, Austria |
| *Glossina palpalis* | IPCL* |  | Institute of Zoology  Slovak Academy of Sciences |

* IPCL = Insect Pest Control Laboratory of the Joint FAO/IAEA Division of Nuclear Techniques in Food and Agriculture, Seibersdorf, Austria

**Supplemental Table 2:** Predicted gene sets for new *Glossina* genomes.

Gene set versions are maintained at ([www.vectorbase.org](http://www.vectorbase.org)) for each organism. All highlighted cells relate to the current gene set version indicated in the table. Statistics for older gene set versions are given along with the relevant version number.

| Species | Initial MAKER protein coding genes (version) | Filtered MAKER protein coding genes (version) | Current gene set version | Predicted RNA genes | Manual gene edits | Total protein coding genes | Total protein coding transcripts |
| --- | --- | --- | --- | --- | --- | --- | --- |
| *G. austeni* | 22,814 (GausT1.1) | 19,763 (GausT1.2) | Gaust1.3 | 483 | 125 | 19,739 | 19,752 |
| *G. brevipalpis* | 19,521 (Gbrel1.1) | 14,627 (Gbrel1.2) | Gbrel1.3 | 371 | 180 | 14,637 | 14,643 |
| *G. fuscipes* | 23,264 (Gfusl1.1) | 20,141 (Gfusl1.2) | Gfusl1.3 | 493 | 199 | 20,129 | 20,145 |
| *G. pallidipes* | 21,935 (Gpall1.1) | 19,282 (Gpall1.2) | Gpall1.3 | 436 | 189 | 19,290 | 19,303 |
| *G. palpalis* | 20,726 (Gpapl1.0) | 20,152 (Gpapl1.0) | Gpapl1.1 | 462 | 90 | 20,153 | 20,158 |

**Supplemental Table 3:** Total measured repetitive elements among Glossina genomes. De novo repeat identification was performed with RepeatModeler [110] and consensus sequences were used in a RepeatMasker [111] analysis which included identification of low complexity/tandem repeats with trf [112] and dust [113]. RepeatMasker output files were filtered to remove redundancy (due to some overlapping hits).

|  | *Glossina morsitans* | *Glossina pallidipes* | *Glossina austeni* | *Glossina fuscipes* | *Glossina palpalis* | *Glossina brevipalpis* |
| --- | --- | --- | --- | --- | --- | --- |
| DNA (Class II) | 6.03% | 6.39% | 6.44% | 5.92% | 5.21% | 7.14% |
| RC (Class II) | 8.69% | 8.36% | 8.65% | 7.49% | 7.53% | 6.00% |
| LINE (Class I) | 5.45% | 5.50% | 5.16% | 5.74% | 5.40% | 2.92% |
| LTR (Class I) | 0.36% | 0.50% | 0.44% | 0.79% | 0.71% | 0.23% |
| SINE (Class I) | 0.11% | 0.09% | 0.06% | 0.04% | 0.04% | 0.00% |
| Unknown | 6.35% | 6.53% | 5.95% | 6.63% | 5.65% | 4.25% |
| Total dispersed (TE) | 26.99% | 27.37% | 26.70% | 26.62% | 24.53% | 20.55% |
| rRNA | 0.00% | 0.00% | 0.02% | 0.03% | 0.02% | 0.00% |
| Simple repeat/Satellite | 7.96% | 8.11% | 11.91% | 10.45% | 15.44% | 17.12% |
| Total | 34.95% | 35.49% | 38.64% | 37.09% | 39.99% | 37.67% |

**Supplemental table 4: TE statistics**

**4a:** Classification of the 7583 RepeatModeler consensuses used in the RepeatMasker analysis. The consensus library of *G. morsitans* initially contained a high number of unclassified sequences which were reclassified through a blastn [120] analysis against the consensus library of *G. pallipides.*

| Subclass | *G. austeni* | *G. brevipalpis* | *G. fuscipes* | *G. morsitans* | *G. pallipides* | *G. palpalis* | Total |
| --- | --- | --- | --- | --- | --- | --- | --- |
| RC (Helitrons) | 85 | 61 | 103 | 60 | 78 | 96 | **483** |
| DNA | 424 | 369 | 377 | 395 | 399 | 353 | **2317** |
| LINE | 309 | 186 | 283 | 304 | 317 | 282 | **1681** |
| LTR | 20 | 8 | 41 | 21 | 23 | 36 | **149** |
| SINE | 3 | 0 | 4 | 4 | 6 | 2 | **19** |
| Unknown | 519 | 305 | 515 | 529 | 473 | 475 | **2816** |
| Simple_Repeat | 11 | 7 | 8 | 14 | 15 | 10 | **65** |
| Other | 1 | 8 | 4 | 3 | 1 | 2 | **53** |
| Total | **1372** | **944** | **1335** | **1364** | **1312** | **1256** | **7583** |

**4b:** Number of consensus clusters by TE subclass and repartition of the coverage according to the number of species containing the cluster. The 7583 consensuses were grouped into 2906 clusters (Usearch5, -id 0.8 -rev) [115]

| Subclass | Number of Clusters | Coverage (in Mb) by clusters present in one to six genomes | | | | | | Total |
| --- | --- | --- | --- | --- | --- | --- | --- | --- |
|  |  | **1** | **2** | **3** | **4** | **5** | **6** |  |
| RC | 261 | 45.42 | 7.5 | 5.43 | 1 | 90.14 | 0 | **149.49** |
| DNA | 825 | 51.66 | 9.67 | 8.69 | 11.42 | 30.86 | 15.69 | **127.99** |
| LINE | 442 | 18.82 | 8.3 | 5.27 | 6.38 | 54.88 | 12.55 | **106.2** |
| LTR | 75 | 5.6 | 0.51 | 0.55 | 0 | 1.8 | 0 | **8.46** |
| SINE | 8 | 0.53 | 0.17 | 0 | 0 | 0 | 0 | **0.7** |
| Unknown | 1152 | 48.52 | 19.01 | 15.42 | 9.46 | 25.16 | 2.36 | **119.93** |
| Ambiguous* | 77 | 2.74 | 11.85 | 3.08 | 2.76 | 14.62 | 0 | **35.05** |
| Simple_repeat | 47 | 2.99 | 0.53 | 0.21 | 0 | 0 | 0 | **3.73** |
| Total | **2887** | **176.28** | **57.54** | **38.65** | **31.02** | **217.46** | **30.6** | **551.55** |

* Ambiguous clusters correspond to clusters in which more than one third of the repeat sequences are differently classified.

**Supplemental Table 5.** Statistics on gene and scaffold mapping to Muller elements.

| Species | genes | mapped genes | % genes mapped | scaffolds | mapped scaffolds | % scaffolds mapped |
| --- | --- | --- | --- | --- | --- | --- |
| *G. austeni* | 19,747 | 6,829 | 34.58% | 671 | 650 | 96.87% |
| *G. brevipalpis* | 14,641 | 6,450 | 44.05% | 448 | 435 | 97.10% |
| *G. fuscipes* | 20,138 | 6,487 | 32.21% | 793 | 764 | 96.34% |
| *G. morsitans* | 12,442 | 6,430 | 51.68% | 1,773 | 1,703 | 96.05% |
| *G. pallidipes* | 19,297 | 6,519 | 33.78% | 544 | 528 | 97.06% |
| *G. palpalis* | 20,160 | 6,322 | 31.36% | 918 | 884 | 96.30% |

**Supplemental table 6.** *Wolbachia* sequences identified in *G. morsitans*, *G. austeni*, *G. fuscipes*, *G. pallidipes*, *G. brevipalpis*, and *G. palpalis*

|  | Cytoplasmic | Chromosomal |
| --- | --- | --- |
| *G. morsitans* |  | 1,013,719 bp |
| *G. pallidipes* | 2,580 bp | No |
| *G. austeni* | 90,846 bp | 960,436 bp |
| *G. palpalis* | 1,050 bp | No |
| *G. fuscipes* | 14,228 bp | No |
| *G. brevipalpis* | 55,476 bp | No |

**Supplemental Table 7: Gene Ontology (GO Molecular Function) Term Enrichment in the *Glossina* Genus and associated sub-Genera**

| GO.ID | Term | Annotated | Significant | Expected | Rank in classic Fisher | Classic Fisher | Elim Fisher | Topgo Fisher | Parentchild Fisher |
| --- | --- | --- | --- | --- | --- | --- | --- | --- | --- |
| Glossina Specific and Universal Enriched GO Terms (Molecular Function) | | | | | | | | | |
| *G. morsitans* | | | | | | | | | |
| GO:0005549 | odorant binding | 78 | 8 | 0.49 | 1 | 2.10E-08 | 2.10E-08 | 2.10E-08 | 1.10E-08 |
| GO:0004252 | serine-type endopeptidase activity | 145 | 7 | 0.91 | 3 | 2.90E-05 | 2.90E-05 | 2.90E-05 | 0.1626 |
| GO:0004190 | aspartic-type endopeptidase activity | 16 | 2 | 0.1 | 8 | 0.0044 | 0.0044 | 0.0044 | 0.1108 |
| *G. pallidipes* | | | | | | | | | |
| GO:0004252 | serine-type endopeptidase activity | 180 | 16 | 1 | 1 | 3.10E-14 | 3.10E-14 | 3.10E-14 | 0.00062 |
| GO:0005549 | odorant binding | 69 | 7 | 0 | 7 | 4.00E-07 | 4.00E-07 | 4.00E-07 | 6.20E-08 |
| GO:0002162 | dystroglycan binding | 1 | 1 | 0 | 10 | 0.0069 | 0.0069 | 0.0069 | 0.00438 |
| *G. austeni* | | | | | | | | | |
| GO:0004252 | serine-type endopeptidase activity | 178 | 17 | 1.24 | 2 | 1.60E-15 | 1.60E-15 | 1.60E-15 | 0.0065 |
| GO:0005549 | odorant binding | 75 | 8 | 0.52 | 7 | 3.90E-08 | 3.90E-08 | 3.90E-08 | 6.90E-09 |
| GO:0004190 | aspartic-type endopeptidase activity | 34 | 4 | 0.24 | 10 | 8.40E-05 | 8.40E-05 | 8.40E-05 | 0.1745 |
| *G. fuscipes* | | | | | | | | | |
| GO:0004252 | serine-type endopeptidase activity | 179 | 19 | 1.09 | 1 | 1.60E-19 | 1.60E-19 | 1.60E-19 | 1.50E-05 |
| GO:0005549 | odorant binding | 79 | 11 | 0.48 | 5 | 8.60E-13 | 8.60E-13 | 8.60E-13 | 2.20E-14 |
| GO:0002162 | dystroglycan binding | 1 | 1 | 0.01 | 10 | 0.0061 | 0.0061 | 0.0061 | 0.0024 |
| *G. palpalis* | | | | | | | | | |
| GO:0004252 | serine-type endopeptidase activity | 177 | 20 | 1.18 | 1 | 2.90E-20 | 2.90E-20 | 2.90E-20 | 5.10E-05 |
| GO:0005549 | odorant binding | 77 | 9 | 0.51 | 7 | 1.40E-09 | 1.40E-09 | 1.40E-09 | 7.20E-11 |
| GO:0003678 | DNA helicase activity | 18 | 2 | 0.12 | 10 | 0.0062 | 0.0062 | 0.0062 | 0.028 |
| *G. brevipalpis* | | | | | | | | | |
| GO:0005549 | odorant binding | 81 | 12 | 0.54 | 1 | 8.50E-14 | 8.50E-14 | 8.50E-14 | 1.00E-14 |
| GO:0004252 | serine-type endopeptidase activity | 163 | 13 | 1.09 | 2 | 2.30E-11 | 2.30E-11 | 2.30E-11 | 0.003 |
| GO:0008036 | diuretic hormone receptor activity | 2 | 2 | 0.01 | 8 | 4.30E-05 | 4.30E-05 | 4.30E-05 | 4.00E-05 |
|  |  |  |  |  |  |  |  |  |  |
| Morsitans sub-genus specific and universal enriched GO Terms (Molecular Function) | | | | | | | | | |
| *G. morsitans* | | | | | | | | | |
| GO:0036402 | proteasome-activating ATPase activity | 10 | 2 | 0.01 | 1 | 6.90E-05 | 6.90E-05 | 6.90E-05 | 0.011 |
| *G. pallidipes* | | | | | | | | | |
| GO:0036402 | proteasome-activating ATPase activity | 9 | 2 | 0.01 | 1 | 2.70E-05 | 2.70E-05 | 2.70E-05 | 0.0074 |
| *G. austeni* | | | | | | | | | |
| GO:0036402 | proteasome-activating ATPase activity | 11 | 3 | 0.02 | 1 | 3.10E-07 | 3.10E-07 | 3.10E-07 | 0.00076 |
|  |  |  |  |  |  |  |  |  |  |
| Palpalis sub-Genus Specific and Universal Enriched GO Terms (Molecular Function) | | | | | | | | | |
| *G. fuscipes* | | | | | | | | | |
| GO:0003677 | DNA binding | 696 | 23 | 11.59 | 4 | 0.00107 | 1.07E-03 | 0.00015 | 0.0476 |
| GO:0004672 | protein kinase activity | 248 | 11 | 4.13 | 13 | 0.00278 | 0.00278 | 0.00048 | 0.0018 |
| GO:0008026 | ATP-dependent helicase activity | 14 | 3 | 0.23 | 7 | 0.00144 | 0.00144 | 0.0016 | 0.0018 |
| *G. palpalis* | | | | | | | | | |
| GO:0004672 | protein kinase activity | 265 | 14 | 4.68 | 9 | 0.00023 | 0.00023 | 5.50E-06 | 0.00011 |
| GO:0003678 | DNA helicase activity | 18 | 3 | 0.32 | 25 | 0.00362 | 0.00362 | 0.00362 | 0.06133 |
| GO:0008026 | ATP-dependent helicase activity | 12 | 2 | 0.21 | 35 | 0.0182 | 0.0182 | 0.0182 | 0.01478 |

**Supplemental Table 8:** Overview of sub-Genus Specific Gene Expansions and Contractions (Numbers represent the number of paralogous sequences per species)

| **VectorBase Gene Tree ID Number** | **Functional Definition** | **Sub-Genus** | **Expansion or Contraction** | ***Glossina brevipalpis*** | ***Glossina austeni*** | ***Glossina morsitans*** | ***Glossina pallidipes*** | ***Glossina palpalis*** | ***Glossina fuscipes*** | **CAFE Derived Family-wide P-value** |
| --- | --- | --- | --- | --- | --- | --- | --- | --- | --- | --- |
| VBGT00190000009793 | 116 kDa U5 small nuclear ribonucleoprotein component | Fusca | Contraction | 5 | 11 | 7 | 8 | 8 | 12 | 0.001 |
| VBGT00190000009794 | laccase-5-like isoform X3 | Fusca | Contraction | 4 | 8 | 5 | 7 | 5 | 7 | 0.011 |
| VBGT00190000009808 | V-type proton ATPase catalytic subunit A | Fusca | Contraction | 6 | 11 | 9 | 11 | 9 | 12 | 0.011 |
| VBGT00190000010180 | zinc finger MIZ domain-containing protein 2 | Fusca | Contraction | 2 | 3 | 5 | 6 | 4 | 6 | 0.004 |
| VBGT00190000011642 | kinesin-like protein KIF20B | Fusca | Contraction | 6 | 13 | 11 | 17 | 13 | 13 | 0.004 |
| VBGT00190000014894 | hypothetical protein | Fusca | Contraction | 1 | 6 | 4 | 4 | 5 | 4 | 0.031 |
| VBGT00190000015811 | hypothetical protein | Fusca | Contraction | 1 | 4 | 5 | 6 | 3 | 6 | 0.004 |
| VBGT00190000016440 | uncharacterized protein | Fusca | Contraction | 2 | 6 | 4 | 6 | 5 | 6 | 0.049 |
| VBGT00190000016667 | hypothetical protein | Fusca | Contraction | 1 | 5 | 2 | 7 | 2 | 4 | 0 |
| VBGT00190000016741 | C3 and PZP-like alpha-2-macroglobulin domain-containing protein | Fusca | Contraction | 1 | 5 | 3 | 5 | 4 | 3 | 0.001 |
| VBGT00750000029331 | hypothetical protein | Fusca | Contraction | 1 | 4 | 3 | 5 | 5 | 5 | 0.028 |
| VBGT00770000031214 | zinc finger protein 699-like | Fusca | Contraction | 10 | 18 | 18 | 18 | 19 | 16 | 0.039 |
| VBGT00820000045940 | transitional endoplasmic reticulum ATPase TER94 | Fusca | Contraction | 20 | 25 | 25 | 21 | 24 | 21 | 0.045 |
| VBGT00820000045950 | activating signal cointegrator 1 complex subunit 3 | Fusca | Contraction | 7 | 13 | 12 | 16 | 15 | 9 | 0 |
| VBGT00820000046004 | dnaJ homolog subfamily A member 4 | Fusca | Contraction | 13 | 19 | 16 | 17 | 15 | 19 | 0.02 |
| VBGT00820000046040 | hypothetical protein | Fusca | Contraction | 2 | 4 | 5 | 6 | 5 | 7 | 0.013 |
| VBGT00840000047885 | Peroxisomal multifunctional enzyme type 2 | Fusca | Contraction | 7 | 11 | 12 | 9 | 8 | 11 | 0.004 |
| VBGT00190000009711 | aldose reductase-like | Fusca | Expansion | 16 | 10 | 8 | 12 | 9 | 11 | 0.008 |
| VBGT00190000009892 | ER degradation-enhancing alpha-mannosidase-like protein 3 | Fusca | Expansion | 11 | 7 | 6 | 4 | 4 | 5 | 0.011 |
| VBGT00190000012881 | GATA zinc finger domain-containing protein 11 | Fusca | Expansion | 13 | 7 | 4 | 7 | 7 | 7 | 0.013 |
| VBGT00190000010664 | trypsin-like serine protease precursor | Morsitans | Contraction | 15 | 5 | 7 | 7 | 14 | 15 | 0.001 |
| VBGT00820000045989 | putative cytochrome P450 28d1 | Morsitans | Contraction | 11 | 7 | 5 | 7 | 14 | 17 | 0 |
| VBGT00190000009926 | lysine-specific demethylase lid | Morsitans | Expansion | 4 | 7 | 7 | 6 | 4 | 3 | 0.036 |
| VBGT00190000010724 | putative cysteine desulfurase, mitochondrial | Morsitans | Expansion | 1 | 4 | 4 | 4 | 1 | 1 | 0.041 |
| VBGT00190000010913 | cytochrome c oxidase subunit 4 isoform 1, mitochondrial | Morsitans | Expansion | 2 | 5 | 5 | 3 | 1 | 2 | 0.001 |
| VBGT00190000010926 | farnesyl pyrophosphate synthase | Morsitans | Expansion | 1 | 5 | 5 | 3 | 1 | 1 | 0.004 |
| VBGT00190000011677 | RUS1 family protein C16orf58 homolog | Morsitans | Expansion | 1 | 5 | 2 | 4 | 1 | 1 | 0.004 |
| VBGT00840000047886 | dehydrogenase/reductase SDR family protein 7-like | Morsitans | Expansion | 7 | 10 | 11 | 10 | 8 | 11 | 0.028 |
| VBGT00190000009725 | Argonaute-1 | Palpalis | Contraction | 13 | 8 | 12 | 12 | 7 | 8 | 0.025 |
| VBGT00190000009849 | valine--tRNA ligase | Palpalis | Expansion | 4 | 7 | 5 | 4 | 7 | 9 | 0.01 |
| VBGT00190000009945 | serine palmitoyltransferase 1 | Palpalis | Expansion | 4 | 6 | 3 | 5 | 12 | 8 | 0 |
| VBGT00190000010218 | TM2 domain-containing protein almondex | Palpalis | Expansion | 1 | 4 | 3 | 3 | 7 | 10 | 0 |
| VBGT00190000010265 | probable phosphorylase b kinase regulatory subunit alpha | Palpalis | Expansion | 2 | 2 | 3 | 2 | 6 | 7 | 0.017 |
| VBGT00190000010375 | mismatch repair endonuclease PMS2 | Palpalis | Expansion | 3 | 2 | 2 | 2 | 6 | 7 | 0.043 |
| VBGT00190000010512 | Zinc transporter foi | Palpalis | Expansion | 1 | 2 | 2 | 3 | 5 | 9 | 0 |
| VBGT00190000010757 | midasin | Palpalis | Expansion | 1 | 4 | 1 | 1 | 7 | 7 | 0 |
| VBGT00190000010980 | Transcription-associated protein 1 | Palpalis | Expansion | 4 | 2 | 2 | 7 | 17 | 13 | 0 |
| VBGT00190000011125 | 2-hydroxyacyl-CoA lyase 1 | Palpalis | Expansion | 1 | 1 | 1 | 1 | 7 | 6 | 0.004 |
| VBGT00190000011176 | pre-mRNA-splicing factor Slu7 | Palpalis | Expansion | 1 | 2 | 2 | 2 | 11 | 12 | 0 |
| VBGT00190000011233 | cytoplasmic FMR1-interacting protein | Palpalis | Expansion | 1 | 1 | 2 | 1 | 6 | 6 | 0.003 |
| VBGT00190000011761 | replication protein A 70 kDa DNA-binding subunit | Palpalis | Expansion | 1 | 3 | 2 | 1 | 5 | 6 | 0.001 |
| VBGT00190000011829 | phosphopantothenoylcysteine decarboxylase | Palpalis | Expansion | 1 | 1 | 1 | 1 | 5 | 6 | 0.015 |
| VBGT00190000012100 | integrator complex subunit 4 | Palpalis | Expansion | 1 | 1 | 1 | 2 | 3 | 5 | 0.004 |
| VBGT00190000012301 | Phosphoglycerate kinase | Palpalis | Expansion | 3 | 3 | 1 | 4 | 5 | 9 | 0 |
| VBGT00190000012418 | phosphoribosylformylglycinamidine synthase | Palpalis | Expansion | 1 | 3 | 1 | 2 | 5 | 4 | 0.003 |
| VBGT00190000012451 | SWI/SNF complex subunit SMARCC2 isoform X2 | Palpalis | Expansion | 2 | 2 | 2 | 3 | 6 | 7 | 0.017 |
| VBGT00190000013378 | hypothetical protein | Palpalis | Expansion | 1 | 1 | 1 | 1 | 4 | 6 | 0.009 |
| VBGT00190000013689 | ATP-dependent DNA helicase 2 subunit 1 | Palpalis | Expansion | 4 | 3 | 2 | 2 | 5 | 7 | 0.011 |
| VBGT00190000013845 | hypothetical protein | Palpalis | Expansion | 1 | 1 | 1 | 1 | 7 | 5 | 0.003 |
| VBGT00190000014141 | WD repeat and HMG-box DNA-binding protein 1 | Palpalis | Expansion | 1 | 1 | 1 | 1 | 5 | 7 | 0.003 |
| VBGT00190000014373 | cilia- and flagella-associated protein 44 | Palpalis | Expansion | 2 | 1 | 1 | 1 | 14 | 10 | 0 |
| VBGT00190000014659 | transmembrane protein 131 homolog | Palpalis | Expansion | 1 | 1 | 1 | 1 | 3 | 6 | 0.003 |
| VBGT00770000031191 | WD repeat-containing protein 78 | Palpalis | Expansion | 4 | 4 | 3 | 4 | 10 | 15 | 0 |
| VBGT00770000031281 | recombination repair protein 1 | Palpalis | Expansion | 1 | 1 | 1 | 1 | 5 | 4 | 0.043 |
| VBGT00770000031399 | Nuclear pore complex protein Nup153 | Palpalis | Expansion | 3 | 3 | 1 | 3 | 4 | 6 | 0.003 |
| VBGT00780000038281 | cell division cycle protein 27 | Palpalis | Expansion | 2 | 3 | 2 | 2 | 14 | 12 | 0 |
| VBGT00840000047928 | hypothetical protein | Palpalis | Expansion | 3 | 4 | 2 | 3 | 8 | 8 | 0.008 |

**Supplemental Table 9: *Glossina* Milk Protein Genes**

| Gene ID | Species | Milk Protein | Scaffold |
| --- | --- | --- | --- |
| GAUT009632 | *G. austeni* | mgp1 | Scaffold14:1466112-1467216:1 |
| GAUT032292 | *G. austeni* | transferrin | Scaffold3:2777421-2780032:-1 |
| GAUT007910 | *G. austeni* | acid sphingomyelinase | Scaffold137:624881-628940:-1 |
| GAUT052949 | *G. austeni* | Mgp2 | Scaffold45:761783:762385:-1 |
| GAUT035464 | *G. austeni* | Mgp3 | Scaffold45:769597:770234:1 |
| GAUT035467 | *G. austeni* | Mgp4 | Scaffold45:758397:758897:1 |
| GAUT035461 | *G. austeni* | Mgp5 | Scaffold45:746356:747009:1 |
| GAUT035463 | *G. austeni* | Mgp6 | Scaffold45:763332:763925:1 |
| GAUT035459 | *G. austeni* | Mgp7 | Scaffold45:736728:737546:1 |
| GAUT035466 | *G. austeni* | Mgp8 | Scaffold45:741110:741706:1 |
| GAUT035460 | *G. austeni* | Mgp9 | Scaffold45:754790:755367:-1 |
| GAUT052948 | *G. austeni* | Mgp10 | Scaffold45:750561:751146:-1 |
| GBRI000512 | *G. brevipalpis* | mgp1 | Scaffold0:865214-866251:1 |
| GBRI004611 | *G. brevipalpis* | transferrin | Scaffold119:130438-132960:-1 |
| GBRI043675 | *G. brevipalpis* | acid sphingomyelinase | Scaffold92:340653-344890:-1 |
| Annotation Pending | *G. brevipalpis* | Mgp3 | Scaffold1:4526048:4525401:-1 |
| GBRI015315 | *G. brevipalpis* | Mgp3 | Scaffold1:4519399:4520451:-1 |
| Annotation Pending | *G. brevipalpis* | Mgp4 | Scaffold1:4533884:4534511:1 |
| GBRI015319 | *G. brevipalpis* | Mgp5 | Scaffold1:4544267:4545482:-1 |
| GBRI015316 | *G. brevipalpis* | Mgp7 | Scaffold1:4552325:4553194:-1 |
| GBRI015317 | *G. brevipalpis* | Mgp8 | Scaffold1:4547741:4549433:-1 |
| GBRI015314 | *G. brevipalpis* | Mgp9 | Scaffold1:4536820:4537630:1 |
| GBRI015312 | *G. brevipalpis* | Mgp10 | Scaffold1:4541300:4541988:1 |
| GFUI006902 | *G. fuscipes* | mgp1 | Scaffold132:123438-124472:1 |
| GFUI040165 | *G. fuscipes* | transferrin | Scaffold57:1030317-1032868:1 |
| GFUI042860 | *G. fuscipes* | acid sphingomyelinase | Scaffold647:119443-123334:1 |
| Annotation Pending partial sequence | *G. fuscipes* | Mgp2 | Scaffold88:162426:162689:1 |
| GFUI050434 | *G. fuscipes* | Mgp3 | Scaffold88:155204:155839:-1 |
| GFUI050428 | *G. fuscipes* | Mgp4 | Scaffold88:166089:166691:1 |
| GFUI050421 | *G. fuscipes* | Mgp5 | Scaffold88:181207:182002:-1 |
| GFUI050422 | *G. fuscipes* | Mgp6 | Scaffold88:159615:161264:-1 |
| GFUI050451 | *G. fuscipes* | Mgp7 | Scaffold88:190717:191338:-1 |
| GFUI050436 | *G. fuscipes* | Mgp8 | Scaffold88:186333:186929:-1 |
| GFUI050420 | *G. fuscipes* | Mgp9 | Scaffold88:170223:170806:1 |
| GFUI050429 | *G. fuscipes* | Mgp10 | Scaffold88:174414:174999:1 |
| GMOY009745 | *G. morsitans* | mgp1 | scf7180000651870:191183-192614:1 |
| GMOY004228 | *G. morsitans* | transferrin | scf7180000648003-44929:47549:1 |
| GMOY002246 | *G. morsitans* | acid sphingomyelinase | scf7180000643139-13861:46922:-1 |
| GMOY001342 | *G. morsitans* | Mgp2 | scf7180000641289:61241:61943:-1 |
| GMOY012125 | *G. morsitans* | Mgp3 | scf7180000641289:68753:72286:1 |
| GMOY012368 | *G. morsitans* | Mgp4 | scf7180000641289:57776:58406:-1 |
| GMOY012370 | *G. morsitans* | Mgp5 | scf7180000641289:42695:43570:1 |
| GMOY001343 | *G. morsitans* | Mgp6 | scf7180000641289:62719:63396:1 |
| GMOY012377 | *G. morsitans* | Mgp7 | scf7180000641289:33723:34473:1 |
| GMOY012016 | *G. morsitans* | Mgp8 | scf7180000641289:38091:38719:1 |
| GMOY012371 | *G. morsitans* | Mgp9 | scf7180000641289:53684:54281:-1 |
| GMOY012369 | *G. morsitans* | Mgp10 | scf7180000641289:49291:50000:-1 |
| GPAI016437 | *G. pallidipes* | mgp1 | Scaffold1:4107747-4108724:-1 |
| GPAI033230 | *G. pallidipes* | transferrin | Scaffold43:693965-696554:-1 |
| GPAI027011 | *G. pallidipes* | acid sphingomyelinase | Scaffold326:50752-54245:-1 |
| GPAI032324 | *G. pallidipes* | Mgp2 | Scaffold41:591935:592530:1 |
| GPAI032303 | *G. pallidipes* | Mgp3 | Scaffold41:584457:585141:-1 |
| GPAI032318 | *G. pallidipes* | Mgp4 | Scaffold41:595496:596201:1 |
| GPAI032322 | *G. pallidipes* | Mgp5 | Scaffold41:609867:610986:-1 |
| GPAI032321 | *G. pallidipes* | Mgp6 | Scaffold41:590355:591123:-1 |
| GPAI032319 | *G. pallidipes* | Mgp7 | Scaffold41:617126:619713:-1 |
| GPAI032320 | *G. pallidipes* | Mgp8 | Scaffold41:614648:615378:-1 |
| GPAI032317 | *G. pallidipes* | Mgp9 | Scaffold41:599399:600206:1 |
| GPAI032316 | *G. pallidipes* | Mgp10 | Scaffold41:603788:604438:1 |
| GPPI015121 | *G. palpalis* | mgp1 | Scaffold68:323777-324973:-1 |
| GPPI043228 | *G. palpalis* | transferrin | Scaffold559:96887-99438:-1 |
| GPPI044202 | *G. palpalis* | acid sphingomyelinase | Scaffold599:29316-34911:-1 |
| GPPI010382 | *G. palpalis* | Mgp2 | Scaffold35:660762:661357:1 |
| GPPI010391 | *G. palpalis* | Mgp3 | Scaffold35:650473:658538:-1 |
| Annotation Pending partial sequence | *G. palpalis* | Mgp4 | Scaffold35:664314:664756:1 |
| GPPI052422 | *G. palpalis* | Mgp5 | Scaffold35:679454:680501:-1 |
| GPPI010392 | *G. palpalis* | Mgp6 | Scaffold35:658759:659920:-1 |
| GPPI052421 | *G. palpalis* | Mgp7 | Scaffold35:688827:689869:-1 |
| GPPI052420 | *G. palpalis* | Mgp8 | Scaffold35:684486:685455:-1 |
| GPPI010383 | *G. palpalis* | Mgp9 | Scaffold35:668551:669134:1 |
| GPPI010380 | *G. palpalis* | Mgp10 | Scaffold35:672681:673401:1 |

**Supplemental Table 10: *Glossina* Male Accessory Protein Gene Orthologs**

| *Glossina morsitans* Seminal Protein Genes | *Glossina pallidipes* Orthologs | *Glossina austeni* Orthologs | *Glossina fuscipes* Orthologs | *Glossina palpalis* Orthologs | *Glossina brevipalpis* Orthologs | Gene description |
| --- | --- | --- | --- | --- | --- | --- |
| GMOY000899 | GPAI014669 | GAUT028232 | GFUI042427 | GPPI014847 | GBRI008346 |  |
| GMOY000930 | GPAI013363 |  |  | GPPI005671 | GBRI027522 | serine protease inhibitor (serpin) 9 [Source:VB Community Annotation] |
| GMOY000990 | GPAI018055 | GAUT027968 | GFUI023610 | GPPI046815 | GBRI042139 | serine protease inhibitor (serpin) 5 [Source:VB Community Annotation] |
| GMOY002262 | GPAI003186 | GAUT020005 | GFUI015527 | GPPI041930 | GBRI036070 | serine protease inhibitor (serpin) 7 [Source:VB Community Annotation] |
| GMOY002279 | GPAI019961 |  |  | GPPI013616 | GBRI033168 |  |
| GMOY002399 | GPAI026065 | GAUT036613 | GFUI045750 | GPPI027571 |  |  |
| GMOY002442 | GPAI039816 | GAUT046193 | GFUI043086 | GPPI017645 | GBRI029243 | serine protease inhibitor (serpin) 1 [Source:VB Community Annotation] |
| GMOY002443 | GPAI039813 | GAUT046196 | GFUI043085 | GPPI017647 | GBRI011243 | serine protease inhibitor (serpin) 2 [Source:VB Community Annotation] |
| GMOY002444 | GPAI039809 | GAUT046192 |  | GPPI017642 | GBRI011241 | serine protease inhibitor (serpin) 3 [Source:VB Community Annotation] |
| GMOY003382 | GPAI030837 | GAUT006156 | GFUI013890 | GPPI025543 | GBRI020025 | serine protease inhibitor (serpin) 11 [Source:VB Community Annotation] |
| GMOY003656 | GPAI039671 | GAUT021607 | GFUI034371 | GPPI048779 | GBRI002517 | serine protease inhibitor (serpin) 4 [Source:VB Community Annotation] |
| GMOY003656 | GPAI039671 | GAUT021607 | GFUI034371 | GPPI048779 | GBRI002522 | serine protease inhibitor (serpin) 4 [Source:VB Community Annotation] |
| GMOY003657 | GPAI039670 | GAUT021609 | GFUI034370 |  | GBRI002518 | serine protease inhibitor (serpin) 5 [Source:VB Community Annotation] |
| GMOY004505 | GPAI006745 | GAUT028678 | GFUI033436 | GPPI037293 | GBRI045069 |  |
| GMOY004506 | GPAI006749 | GAUT028675 | GFUI033435 | GPPI021164 |  |  |
| GMOY004724 | GPAI035555 | GAUT022204 | GFUI052930 | GPPI025518 |  |  |
| GMOY004725 |  | GAUT022209 |  |  | GBRI034889 |  |
| GMOY004726 | GPAI035537 | GAUT022203 |  |  | GBRI034889 |  |
| GMOY004727 |  | GAUT022202 |  |  | GBRI034889 |  |
| GMOY004969 | GPAI009113 |  | GFUI048292 | GPPI031227 | GBRI045069 |  |
| GMOY005771 | GPAI012918 | GAUT025963 |  |  | GBRI005448 |  |
| GMOY005874 | GPAI017999 | GAUT029311 | GFUI008562 | GPPI028532 | GBRI010920 |  |
| GMOY005875 | GPAI018000 | GAUT029310 | GFUI008563 | GPPI028531 | GBRI010919 |  |
| GMOY005876 | GPAI018009 | GAUT029308 | GFUI008564 | GPPI028521 | GBRI010929 |  |
| GMOY005876 | GPAI018009 | GAUT029308 | GFUI008564 | GPPI028521 | GBRI010924 |  |
| GMOY006016 |  | GAUT021504 | GFUI051598 | GPPI013618 | GBRI033168 | serine proteinase inhibitor [Source:VB Community Annotation] |
| GMOY006927 | GPAI037620 | GAUT021884 | GFUI021198 | GPPI018038 | GBRI023575 |  |
| GMOY006928 | GPAI037622 | GAUT021882 | GFUI021195 | GPPI018036 | GBRI023572 |  |
| GMOY007314 | GPAI006440 | GAUT028974 | GFUI007906 | GPPI023932 | GBRI036202 | odorant binding protein 17 [Source:VB Community Annotation] |
| GMOY007757 | GPAI008777 | GAUT040992 | GFUI008988 | GPPI008631 | GBRI016436 | odorant binding protein 4 [Source:VB Community Annotation] |
| GMOY007759 | GPAI008749 | GAUT040977 | GFUI008965 | GPPI052283 | GBRI016473 |  |
| GMOY007760 | GPAI008750 | GAUT005680 |  |  |  |  |
| GMOY008627 | GPAI041316 | GAUT048422 | GFUI011319 | GPPI037728 |  |  |
| GMOY008628 | GPAI041313 | GAUT048423 | GFUI011316 | GPPI037724 | GBRI040455 |  |
| GMOY008942 | GPAI029944 | GAUT039250 | GFUI015916 | GPPI027927 | GBRI008095 | serine protease inhibitor (serpin) 6 [Source:VB Community Annotation] |
| GMOY009481 | GPAI041306 | GAUT044880 | GFUI011310 |  |  |  |
| GMOY009777 |  | GAUT012797 |  | GPPI028287 | GBRI032716 |  |
| GMOY009778 | GPAI004192 |  | GFUI008929 | GPPI028285 |  |  |
| GMOY010053 | GPAI020254 | GAUT051462 | GFUI012429 | GPPI016282 | GBRI025356 | serine protease inhibitor (serpin) 12 [Source:VB Community Annotation] |
| GMOY012007 | GPAI011576 | GAUT014044 | GFUI019208 | GPPI033766 | GBRI004383 | serine protease inhibitor (serpin) 10 [Source:VB Community Annotation] |
| GMOY012229 | GPAI008752 | GAUT052951 | GFUI054345 | GPPI052451 | GBRI016471 |  |
| GMOY012237 | GPAI006441 | GAUT028973 | GFUI007905 | GPPI023929 | GBRI036200 |  |
| GMOY012237 | GPAI006441 | GAUT028973 | GFUI007905 | GPPI023929 | GBRI036198 |  |
| GMOY013003 | GPAI041312 | GAUT048424 | GFUI011318 |  | GBRI040454 |  |
| GMOY013035 | GPAI018055 | GAUT027968 | GFUI023610 | GPPI046815 | GBRI042139 | Serine protease inhibitor (serpin) 5 [Source:UniProtKB/TrEMBL;Acc:A0A1B0GGD0] |
| GMOY013054 | GPAI049061 | GAUT028235 | GFUI042426 | GPPI014848 | GBRI045553 |  |
| GMOY013055 | GPAI014675 | GAUT028233 | GFUI042425 | GPPI002594 | GBRI008349 |  |
| GMOY013055 | GPAI014675 | GAUT028233 | GFUI042425 | GPPI052269 | GBRI008349 |  |
| GMOY013075 | GPAI041873 |  | GFUI038743 | GPPI041861 | GBRI005448 |  |
| GMOY013229 | GPAI001679 | GAUT015080 | GFUI038739 | GPPI041864 | GBRI005448 |  |
| GMOY013305 |  | GAUT052823 | GFUI038747 | GPPI041862 | GBRI005448 |  |
| GMOY013306 |  | GAUT052825 |  |  | GBRI005448 |  |
| GMOY013307 | GPAI041874 | GAUT052824 | GFUI038743 | GPPI041861 | GBRI005448 | Putative uncharacterized protein [Source:UniProtKB/TrEMBL;Acc:D3TQH3] |
| GMOY013308 |  |  |  |  | GBRI005448 |  |

**Supplemental Table 11:** Neuropeptide genes identified in various tsetse flies, including *Glossina morsitans*, as deduced by homology searches of the respective genomes *in silico*. The gene identification number is supplied, or listed as “not located” when not found. Where more than one gene is identified in one species and not in another, the latter is indicated as “no” for no other gene identified. Rows colored in brown are lacking representation across all *Glossina* species while yellow rows represent partial representation

| **Neuropeptide^1^** | ***G.morsitans*** | ***G. pallidipes*** | ***G. austeni*** | ***G. palpalis*** | ***G. fuscipes*** | ***G. brevipalpis*** |
| --- | --- | --- | --- | --- | --- | --- |
| ACP | not located | not located | not located | not located | not located | not located |
| Adipokinetic hormone | GMOY003470 | GPAI036121 | GAUT013261 | GPPI030617 | GFUI054167 | GBRI027509 |
|  | GMOY003469 | GPAI049064 | GAUT013267 | GPPI030614 | GFUI054166 | GBRI045557 |
| Allatotropin | not located | not located | not located | not located | not located | not located |
| Allatostatin A | GMOY002621 | GPAI037922 | GAUT042383 | GPPI044384 | GFUI015852 | GBRI014005 |
| Allatostatin B | GMOY012235 | GPAI046483 | GAUT051974 | GPPI036833 | GFUI028593 | GBRI016323 no confirmation now. |
| Allatostatin C | GMOY012106 | GPAI030796 | GAUT018803 | GPPI008165 | GFUI031621 | GBRI013358 |
|  | no | no | GAUT018801 | no | no | no |
| Bursicon-α | GMOY011521 | GPAI045899 | GAUT041352 | GPPI024687 | GFUI031519 | GBRI004157 |
| Bursicon-β | GMOY004325 | GPAI005625 | GAUT008609 | GPPI032353 | GFUI051829 | GBRI001434 |
|  | no | no | no | GPPI047490 | GFUI051824 | GBRI001449 |
| Capa/Pyrokinin | GMOY003803 | GPAI048036 | GAUT035627 | GPPI042459 | GFUI023068 | GBRI037790 |
| CCAP | GMOY009420 | GPAI014903 | GAUT031720 | GPPI035716 | GFUI009406 | GBRI021868 |
| CCHamide-1 | GMOY003581 | GPAI017333 | GAUT048738 | GPPI050261 | GFUI047419 | not located |
|  | no | no | GAUT000929 | no | no | no |
| CCHamide-2 | GMOY010764 | GPAI006272 | GAUT047058 | GPPI027237 | GFUI034473 | GBRI028417 |
| Corazonin | GMOY012060 | GPAI024124 | GAUT003311 | GPPI019152 | GFUI053499 | GBRI045556 |
| DH (calcitonin) | GMOY003573 | GPAI011088 | GAUT009941 | GPPI036049 | GFUI008406 | GBRI018412: not verified |
| DH (CRF) | GMOY012107 | GPAI020401 | GAUT031801 | GPPI047047 | GFUI034251 | GBRI021944 |
| EH | GMOY012086 | not located | not located | GPPI046027 | not located | GBRI026411 |
| ETH | GMOY012358 | GPAI036755 | GAUT019585 | GPPI024407 | GFUI016014 | GBRI013026: not confirmed |
| FMRFamide | GMOY003883 | GPAI028872 | GAUT023561 | GPPI011945 | GFUI022977 | GBRI025526 |
| Glycoprotein α-2 | GMOY006315 | GPAI012868 | GAUT035273 | GPPI035304 | GFUI018308 | GBRI007065 |
| Glycoprotein β-5 | GMOY006313 | GPAI012869 | GAUT035261 | GPPI035289 | GFUI018328 | GBRI007062 |
| Hugin | GMOY003497 | GPAI029434 | GAUT035879 | GPPI016885 | GFUI053332 | GBRI037427 |
| ILP | GMOY003945 | GPAI002313 | GAUT046586 | GPPI022721 | GFUI049575 | GBRI018552 |
|  | GMOY003946 | GPAI001883 | GAUT006766 | GPPI015490 | GFUI041241 | GBRI033759 |
|  | GMOY005180 | no | no | no | no | GBRI004816 |
|  | GMOY002258 | no | no | no | no | no |
|  | GMOY006879 | no | no | no | no | no |
| Inotocin | not located | not located | not located | not located | not located | not located |
| ITP | GMOY012155 | GPAI043749 | GAUT047668 | GPPI029894 | GFUI042528 | GBRI044413 |
| Leucokinin | GMOY012112 | GPAI044412 | GAUT037790 | GPPI015558 | GFUI005571 | GBRI009975: not confirmed |
| Myosuppressin | GMOY011892 | GPAI035646 | GAUT034236 | GPPI027780 | GFUI041280 | GBRI017368 |
| Natalisin | GMOY006483 | GPAI024429 | GAUT036435 | GPPI022174 | GFUI014204 | GBRI016581 |
| Neuroparsin/crimpy | GMOY011055 | GPAI027170 | GAUT026343 | GPPI005114 | GFUI045135 | GBRI013110 |
| NPF | GMOY012182 | not located | GAUT035914 | GPPI016939 | GFUI053299 | not located |
| NPLP-1 | GMOY005894 | GPAI004353 | GAUT049217 | GPPI029397 | GFUI033879 | GBRI033649 |
| Orcokinin | GMOY009230 | GPAI025930 | GAUT027488 | GPPI023490 | GFUI026883 | GBRI002434 |
| PDF | GMOY000603 | GPAI046761 | GAUT031282 | GPPI022815 | GFUI049486 | not located |
| Proctolin | GMOY012104 | GPAI007711 | GAUT023108 | GPPI011465 | GFUI031914 | GBRI000660 |
| PTTH | GMOY011991 | GPAI007078 | GAUT033621 | GPPI021954 | GFUI025672 | GBRI015505 |
| RYamide | not located | not located | GAUT023352 | GPPI019391 | GFUI018333 | GBRI044269 |
| Short NPF | GMOY012142 | GPAI020549 | GAUT011332 | GPPI043755 | GFUI032056 | GBRI022854 |
| SIFamide | GMOY005296 | GPAI045409 | GAUT032331 | GPPI020456 | GFUI039970 | GBRI045710 |
|  | no | GPAI033266 | no | GPPI014368 | GFUI040137 | GBRI004649 |
| Sulfakinin | not located | not located | not located | not located | not located | not located |
| Tachykinin | GMOY008567 | GPAI020386 | GAUT031793 | GPPI048084 | GFUI034255 | GBRI021939 |
| Trissin | GMOY009417 | GPAI014911 | GAUT031727 | GPPI035715 | GFUI009405 | GBRI021869 |

^1^Abbreviated peptide names: ACP, adipokinetic hormone/corazonin-like neuropeptide; CCAP, crustacean cardioactive peptide; DH (calcitonin), calcitonin-like diuretic hormone; DH (CRF), corticotropin releasing factor-like diuretic hormone; EH, eclosion hormone; ETH, ecdysis triggering hormone; ILP, insulin-like peptide; ITP, ion-transporting peptide; NPF, neuropeptide F; NPLP, neuropeptide-like precursor; PDF, pigment-dispersing factor; PTTH, prothoracicotropic hormone.

CNMamide [Source:Projected from *Drosophila melanogaster* (FBgn0035282) FlyBase gene name;Acc:FBgn0035282]; *Musca domestica* = MDOA011180 & *Stomoxys calcitrans* = SCAU014405. Not in *Glossina spp* nor in mosquitoes.

**Supplemental Table 12:** Protein receptor genes for bioactive neuropeptides identified in various tsetse flies, including *Glossina morsitans*, as well as the common housefly *Musca domestica*, as deduced by homology searches of the respective genomes *in silico*. The gene identification number is supplied, or listed as “not located” when not found. Where more than one gene is identified in one species and not in another, the latter is indicated as “no” for no other gene identified.

| **Protein Receptor for^1^** | ***G. morsitans*** | ***G. pallidipes*** | ***G. austeni*** | ***G. palpalis*** | ***G. fuscipes*** | ***G. brevipalpis*** | ***M. domestica*** |
| --- | --- | --- | --- | --- | --- | --- | --- |
| ACP | not located | not located | not located |  | not located | not located | not located |
| Adipokinetic hormone | GMOY008368 | GPAI026595 | GAUT019061 | GPPI021443 | GFUI027776 | GBRI000966 | MDOA004853 |
| Allatotropin | not located | not located | not located | not located | not located | not located | not located |
| Allatostatin A | GMOY012001 | GPAI029482 | GAUT035837 | GPPI017451 | GFUI053372 | GBRI037476 | MDOA015456 |
|  | GMOY005179 | GPAI010186 | GAUT000491 | GPPI037221 | GFUI036826 | GBRI033753 | MDOA007526 |
| Allatostatin B | not located | not located | not located | not located | not located | not located | not located |
| Allatostatin C | GMOY002651 | GPAI046718 | GAUT041867 | GPPI012370 | GFUI036377 | GBRI004988 | MDOA007493 |
|  | GMOY012068 | GPAI019597 | no | GPPI032911 | GFUI036404 | GBRI004982 | MDOA005747 |
| Bursicon-α | GMOY011521 | GPAI045899 | not located | GPPI024687 | GFUI031519 | GBRI004157 | MDOA000854 |
|  | GMOY003161 | GPAI014560 | GAUT050652 | GPPI014972 | GFUI006325 | GBRI032052 | MDOA005834 |
| Bursicon-β | GMOY004325 | GPAI005625 | not located | GPPI032353 | GFUI051829 | not located | MDOA014861 |
|  | no | no | no | GPPI047490 | no | no | no |
| Capa /Cap2B/ Pyrokinin/Hugin | GMOY012161 | GPAI046497 | GAUT051954 | GPPI036820 | GFUI028612 | not located | MDOA015286 |
|  | GMOY012119 | GPAI017332 | GAUT018604 | GPPI050255 | GFUI047418 | GBRI031007 | MDOA014609 |
|  | GMOY009301 | GPAI045130 | GAUT048737 | no | GFUI035668 | GBRI032091 | MDOA010837 |
|  | no | no | GAUT051726 | no | no | no | no |
| CCAP | GMOY009062 | GPAI039220 | GAUT010112 | GPPI038279 | GFUI046430 | GBRI009844 | MDOA013475 |
| CCHamide-1 | GMOY006903 | GPAI028941 | GAUT030352 | GPPI040785 | GFUI016889 | GBRI002327 | MDOA013652 |
| CCHamide-2 | GMOY009111 | GPAI044844 | GAUT023072 | GPPI016208 | GFUI015341 | GBRI028932 | MDOA013613 |
| CNMamide | GMOY005917 | GPAI017658 | GAUT003612 | GPPI043177 | GFUI025643 | GBRI031527 | MDOA000808 |
|  | no | no | no | no | GFUI027056 | no | MDOA016759 |
| Corazonin | GMOY006527 | GPAI013494 | GAUT015004 | not located | GFUI040669 | GBRI016365 | MDOA014451 |
|  | no | no | no | no | no | no | MDOA008078 |
| DH 31 (calcitonin) | GMOY010919 | GPAI028216 | GAUT023385 | GPPI048644 | GFUI017241 | GBRI044319 | MDOA000230 |
| DH 44 (CRF) | GMOY012160 | GPAI034419 | GAUT043673 | GPPI028529 | GFUI008579 | GBRI044266 | MDOA009175 |
|  | GMOY012023 | GPAI017992 | GAUT029316 | GPPI028527 | GFUI008577 | GBRI010922 | MDOA006927 |
|  | no | GPAI027700 | GAUT023363 | GPPI043324 | GFUI017282 | no | MDOA006619 |
| ETH | GMOY012065 | GPAI007847 | GAUT007145 | GPPI045513 | GFUI000307 | GBRI036878 | MDOA002914 |
| FMRFamide | GMOY012135 | not located | not located | GPPI020871 | GFUI044848 | not located | MDOA002590 |
| Glycoprotein 2α / 5β | GMOY008940 | GPAI038760 | GAUT035203 | GPPI015990 | GFUI026094 | GBRI013835 | MDOA014289 |
|  | GMOY006337 | no | no | no | no | no | no |
| Hector | GMOY009447 | GPAI031364 | GAUT050139 | GPPI012503 | GFUI051144 | not located | MDOA006082 |
|  | no | no | GAUT050138 | no | no | no | no |
| Leucokinin | GMOY007765 | GPAI032398 | GAUT035408 | GPPI047278 | not located | GBRI042364 | MDOA009703 |
| Lgr3/Orphan GPCR 6 | GMOY012010 | GPAI005664 | GAUT009324 | GPPI021840 | GFUI017878 | GBRI041157 | MDOA002739 |
| Moody | GMOY003467 | GPAI021938 | GAUT014222 | GPPI025997 | GFUI045655 | GBRI045038 | MDOA001450 |
| Myosuppressin | GMOY009670 | GPAI005116 | GAUT047489 | GPPI008887 | GFUI034109 | GBRI039573 | MDOA003714 |
|  | no | no | no | no | no | no | MDOA012230 |
| NPF | GMOY006767 | GPAI023998 | GAUT038865 | GPPI019011 | GFUI050788 | GBRI007095 | MDOA001172 |
| Orphan GPCR 2 | GMOY001972 | not located | GAUT015610 | not located | GFUI047692 | GBRI034114 | MDOA006597 |
| Orphan GPCR 3 | GMOY005600 | GPAI021925 | GAUT014227 | GPPI025988 | GFUI045672 | GBRI045041 | MDOA002331 |
| Orphan GPCR 7 | GMOY012103 | GPAI034951 | GAUT027762 | GPPI009196 | GFUI024162 | GBRI044968 | MDOA005523 |
| Orphan GPCR 8 | GMOY011869 | GPAI020655 | GAUT008659 | GPPI034431 | GFUI004073 | GBRI012070 | MDOA009098 |
| Orphan GPCR 9 | GMOY012305 | GPAI024566 | GAUT033522 | GPPI044476 | GFUI027275 | GBRI012466 | MDOA007874 |
| Orphan GPCR 10 | GMOY012043 | GPAI001438 | GAUT021415 | GPPI029024 | GFUI034321 | GBRI003919 | MDOA005504 |
| PDF | GMOY007422 | GPAI031267 | GAUT050246 | GPPI002487 | GFUI003580 | GBRI010392 | MDOA001908 |
| Proctolin | GMOY011680 | GPAI048197 | GAUT015414 | GPPI001122 | GFUI001648 | GBRI003761 | MDOA010087 |
| RYamide/mammalian NPY-like | GMOY011997 | GPAI021450 | GAUT014810 | GPPI032837 | GFUI043225 | GBRI000226 | MDOA001259 |
|  | GMOY012052 | GPAI021430 | GAUT014822 | GPPI017273 | GFUI043227 | GBRI000235 | MDOA012402 |
| MIP Receptor | GMOY004860 | GPAI042600 | GAUT040731 | GPPI002955 | GFUI026358 | GBRI023627 | MDOA006144 |
|  | no | no | no | no | GFUI026351 | no | no |
| Short NPF | GMOY006636 | not located | GAUT037859 | GPPI019355 | GFUI005605 | GBRI010003 | MDOA011011 |
| SIFamide | GMOY008798 | GPAI007832 | GAUT007169 | GPPI044149 | GFUI047709 | GBRI036855 | MDOA007765 |
| Sulfakinin | not located | not located | not located | not located | not located | GBRI027745 | MDOA011233 |
|  | no | no | no | no | no | GBRI027746 | MDOA008617 |
| Tachykinin (Putative Natalisin Receptor?) | GMOY011823 | GPAI019001 | GAUT046129 | GPPI009923 | GFUI038255 | GBRI012822 | MDOA014354 |
|  | GMOY004521 | no | no | no | no | no | no |
| Trissin | GMOY010450 | GPAI037343 | GAUT022572 | GPPI034168 | GFUI034351 | GBRI035755 | MDOA015069 |

^1^Abbreviated peptide names: ACP, adipokinetic hormone/corazonin-like neuropeptide; CCAP, crustacean cardioactive peptide; DH (calcitonin), calcitonin-like diuretic hormone; DH (CRF), corticotropin releasing factor-like diuretic hormone; EH, eclosion hormone; ETH, ecdysis triggering hormone; ILP, insulin-like peptide; ITP, ion-transporting peptide; NPF, neuropeptide F; NPLP, neuropeptide-like precursor; PDF, pigment-dispersing factor; PTTH, prothoracicotropic hormone. NPY neuropeptide Y-like

CNMamide Receptor *D. melanogaster* annotation symbol CG33696, Flybase ID FBgn0053696, **CNMamide** (**CNMa**) is a cyclic [neuropeptide](https://en.wikipedia.org/wiki/Neuropeptide) identified by [computational analysis](https://en.wikipedia.org/w/index.php?title=Computational_analysis&action=edit&redlink=1) of [Drosophila melanogaster](https://en.wikipedia.org/wiki/Drosophila_melanogaster) protein sequences and named after its C-terminal ending motif. A gene encoding CNMa was found in most [arthropods](https://en.wikipedia.org/wiki/Arthropod) and comparison among the precursor sequences of several representative species revealed high conservation, particularly in the region of the predicted mature peptide.[^[1]^](https://en.wikipedia.org/wiki/CNMa#cite_note-cnma-1) Two conserved [cysteine](https://en.wikipedia.org/wiki/Cysteine) residues enveloping four amino acids form a [disulfide bond](https://en.wikipedia.org/wiki/Disulfide_bond) and were shown to be important for binding of the peptide to its receptor. Expression of CNMa was confirmed in the larval and adult brain of D. melanogaster but the function of the peptide has not been elucidated yet.

Hector is a G-protein coupled receptor involved in male courtship behavior. *Drosophila melanogaster*: CG4395; FBgn0030437.

Lgr3 = Leucine-rich repeat-containing G protein-coupled receptor 3; FBgn0039354

*Drosophila melanogaster*: moody (FBgn0025631), annotation symbol CG4322. For ventral nerve cord development, Isoform A and isoform B are required in glia to regulate the acute sensitivity to cocaine and to continuously maintain the proper blood-brain barrier (BBB) function. A moody-mediated signaling pathway functions in glia to regulate nervous system insulation and drug-related behaviors. Galphai and Galphao, and the regulator of G protein signaling, loco, are required in the surface glia to achieve effective.

**Supplemental Table 13. Family specific counts of putative cuticle protein genes across *Glossina* genomes.**

|  | CPR^a^ | | |  |  |  |  |  |  |  |  |
| --- | --- | --- | --- | --- | --- | --- | --- | --- | --- | --- | --- |
|  | RR-1 | RR-2 | RR-Uncl | CPAP1 | CPAP3 | CPF | CPCFC | CPLCA | CPLCG | TWDL | Total |
| *G. austeni* | 23 | 23 | 14 | 12 | 4 | 1 | 1 | 6 | 5 | 12 | 101 |
| *G. brevipalpis* | 33 | 25 | 16 | 10 | 5 | 1 | 1 | 7 | 5 | 10 | 113 |
| *G. fuscipes* | 37 | 28 | 20 | 13 | 5 | 1 | 1 | 7 | 5 | 13 | 130 |
| *G. pallidipes* | 34 | 27 | 19 | 11 | 6 | 1 | 1 | 9 | 4 | 10 | 122 |
| *G. palpalis* | 36 | 26 | 18 | 10 | 4 | 1 | 2 | 8 | 5 | 8 | 118 |

^a^Sequences that scored above the assigned cutoffs for the RR-1 and RR-2 models were classified as the corresponding type, whereas sequences with scores below the assigned cutoffs but above 0 were characterized as “unclassified” (Uncl). For more information, see [121].

**Supplemental Table 14.** List of all peritrophin and peritrophin-like proteins (PLPs), containing one or more chitin binding domains (CBDs) across the six major *Glossina* species, and their orthologues in *Drosophila*.

Suggested gene names are those shown under the heading ‘symbol and synonym’.

*Drosophila* genes highlighted in yellow are those that have been shown to be constitutes of the peritrophic matrix (GO:0016490) either through experimental evidence or by electronic inference.

| FlyBase ID | VectorBase ID | Species | Symbol | Synonym | Description |
| --- | --- | --- | --- | --- | --- |
| FBgn0052036 | GMOY000880 | Morsitans | GmmPLP1 | Peritrophin-Like Protein | Chitin-binding, Peritrophin-A-Domain |
|  | GAUT003677 | Austeni | GauPLP1 | Peritrophin-Like Protein | Chitin-binding, Peritrophin-A-Domain |
|  | GBRI031179 | Brevipalpis | GbrPLP1 | Peritrophin-Like Protein | Chitin-binding, Peritrophin-A-Domain |
|  | GFUI027404 | Fuscipes | GfuPLP1 | Peritrophin-Like Protein | Chitin-binding, Peritrophin-A-Domain |
|  | GPAI017590 | Palldipes | GpaPLP1 | Peritrophin-Like Protein | Chitin-binding, Peritrophin-A-Domain |
|  | GPPI025392 | Palpalis | GppPLP1 | Peritrophin-Like Protein | Chitin-binding, Peritrophin-A-Domain |
|  |  |  |  |  |  |
| FBgn0052024 | GMOY000968 | Morsitans | GmmPLP2 | Peritrophin-Like Protein | Chitin-binding, Peritrophin-A-Domain |
|  | GAUT010978 | Austeni | GauPLP2 | Peritrophin-Like Protein | Chitin-binding, Peritrophin-A-Domain |
|  | GBRI005049 | Brevipalpis | GbrPLP2 | Peritrophin-Like Protein | Chitin-binding, Peritrophin-A-Domain |
|  | GFUI013195 | Fuscipes | GfuPLP2 | Peritrophin-Like Protein | Chitin-binding, Peritrophin-A-Domain |
|  | GPAI009989 | Palldipes | GpaPLP2 | Peritrophin-Like Protein | Chitin-binding, Peritrophin-A-Domain |
|  | GPPI003158 | Palpalis | GppPLP1 | Peritrophin-Like Protein | Chitin-binding, Peritrophin-A-Domain |
|  |  |  |  |  |  |
| FBgn0030999 | GMOY001312 | Morsitans | GmmPLP3 | Peritrophin-Like Protein | Chitin-binding, Peritrophin-A-Domain |
|  | GAUT020830 | Austeni | GauPLP3 | Peritrophin-Like Protein | Chitin-binding, Peritrophin-A-Domain |
|  | GBRI007737 | Brevipalpis | GbrPLP3 | Peritrophin-Like Protein | Chitin-binding, Peritrophin-A-Domain |
|  | GFUI048226 | Fuscipes | GfuPLP3 | Peritrophin-Like Protein | Chitin-binding, Peritrophin-A-Domain |
|  | GPAI009178 | Palldipes | GpaPLP3 | Peritrophin-Like Protein | Chitin-binding, Peritrophin-A-Domain |
|  |  |  |  |  |  |
| FBgn0035430 | GMOY001522 | Morsitans | GmmPLP4 | Peritrophin-Like Protein | Chitin-binding, Peritrophin-A-Domain |
|  | GAUT026255 | Austeni | GauPLP4 | Peritrophin-Like Protein | Chitin-binding, Peritrophin-A-Domain |
|  | GBRI023956 | Brevipalpis | GbrPLP4 | Peritrophin-Like Protein | Chitin-binding, Peritrophin-A-Domain |
|  | GFUI039257 | Fuscipes | GfuPLP4 | Peritrophin-Like Protein | Chitin-binding, Peritrophin-A-Domain |
|  | GPAI027229 | Palldipes | GpaPLP4 | Peritrophin-Like Protein | Chitin-binding, Peritrophin-A-Domain |
|  | GPPI005038 | Palpalis | GppPLP4 | Peritrophin-Like Protein | Chitin-binding, Peritrophin-A-Domain |
|  |  |  |  |  |  |
|  | GMOY001662 | Morsitans | GmmPLP5a | Peritrophin-Like Protein | Chitin-binding, Peritrophin-A-Domain |
|  | GMOY002339 | Morsitans | GmmPLP5b | Peritrophin-Like Protein | Chitin-binding, Peritrophin-A-Domain |
|  | GAUT014249 | Austeni | GauPLP5 | Peritrophin-Like Protein | Chitin-binding, Peritrophin-A-Domain |
|  | GBRI000180 | Brevipalpis | GbrPLP5 | Peritrophin-Like Protein | Chitin-binding, Peritrophin-A-Domain |
|  | GFUI007094 | Fuscipes | GfuPLP5a | Peritrophin-Like Protein | Chitin-binding, Peritrophin-A-Domain |
|  | GFUI016406 | Fuscipes | GfuPLP5b | Peritrophin-Like Protein | Chitin-binding, Peritrophin-A-Domain |
|  | GPAI001697 | Palldipes | GpaPLP5a | Peritrophin-Like Protein | Chitin-binding, Peritrophin-A-Domain |
|  | GPAI021942 | Palldipes | GpaPLP5b | Peritrophin-Like Protein | Chitin-binding, Peritrophin-A-Domain |
|  |  |  |  |  |  |
| FBgn0038632 | GMOY002084 | Morsitans | GmmPLP6 | Peritrophin-Like Protein | Chitin-binding, Peritrophin-A-Domain |
|  | GAUT027066 | Austeni | GauPLP6 | Peritrophin-Like Protein | Chitin-binding, Peritrophin-A-Domain |
|  | GBRI004278 | Brevipalpis | GbrPLP6 | Peritrophin-Like Protein | Chitin-binding, Peritrophin-A-Domain |
|  | GFUI036017 | Fuscipes | GfuPLP6 | Peritrophin-Like Protein | Chitin-binding, Peritrophin-A-Domain |
|  | GPAI012018 | Palldipes | GpaPLP6 | Peritrophin-Like Protein | Chitin-binding, Peritrophin-A-Domain |
|  |  |  |  |  |  |
| FBgn0038629 | GMOY002086 | Morsitans | GmmPLP7 | Peritrophin-Like Protein | Chitin-binding, Peritrophin-A-Domain |
|  | GAUT027081 | Austeni | GauPLP7 | Peritrophin-Like Protein | Chitin-binding, Peritrophin-A-Domain |
|  | GBRI004277 | Brevipalpis | GbrPLP7 | Peritrophin-Like Protein | Chitin-binding, Peritrophin-A-Domain |
|  | GPPI029843 | Palpalis | GppPLP7 | Peritrophin-Like Protein | Chitin-binding, Peritrophin-A-Domain |
|  |  |  |  |  |  |
| FBgn0037488 | GMOY002141 | Morsitans | GmmPLP8 | Peritrophin-Like Protein | Chitin-binding, Peritrophin-A-Domain |
|  | GAUT044450 | Austeni | GauPLP8 | Peritrophin-Like Protein | Chitin-binding, Peritrophin-A-Domain |
|  | GBRI023660 | Brevipalpis | GbrPLP8 | Peritrophin-Like Protein | Chitin-binding, Peritrophin-A-Domain |
|  | GFUI027472 | Fuscipes | GfuPLP8a | Peritrophin-Like Protein | Chitin-binding, Peritrophin-A-Domain |
|  | GFUI048634 | Fuscipes | GfuPLP8b | Peritrophin-Like Protein | Chitin-binding, Peritrophin-A-Domain |
|  | GPAI005401 | Palldipes | GpaPLP8 | Peritrophin-Like Protein | Chitin-binding, Peritrophin-A-Domain |
|  | GPPI048548 | Palpalis | GppPLP8 | Peritrophin-Like Protein | Chitin-binding, Peritrophin-A-Domain |
|  |  |  |  |  |  |
| FBgn0037487 | GMOY002142 | Morsitans | GmmPLP9 | Peritrophin-Like Protein | Chitin-binding, Peritrophin-A-Domain |
|  |  |  |  |  |  |
| FBgn0263748 | GMOY002708 | Morsitans | GmmPLP10  GmmPer66 | Peritrophin-Like Protein/GmmPer66 | Chitin-binding, Peritrophin-A-Domain, mucin domains |
| FBgn0085353 | GAUT001531 | Austeni | GauPLP10a | Peritrophin-Like Protein | Chitin-binding, Peritrophin-A-Domain, mucin domains |
|  | GAUT001532 | Austeni | GauPLP10b | Peritrophin-Like Protein | Chitin-binding, Peritrophin-A-Domain, mucin domains |
|  | GBRI011740 | Brevipalpis | GbrPLP10a | Peritrophin-Like Protein | Chitin-binding, Peritrophin-A-Domain, mucin domains |
|  | GBRI011741 | Brevipalpis | GbrPLP10b | Peritrophin-Like Protein | Chitin-binding, Peritrophin-A-Domain, mucin domains |
|  | GFUI006263 | Fuscipes | GfuPLP10a | Peritrophin-Like Protein | Chitin-binding, Peritrophin-A-Domain, mucin domains |
|  | GFUI006267 | Fuscipes | GfuPLP10b | Peritrophin-Like Protein | Chitin-binding, Peritrophin-A-Domain, mucin domains |
|  | GPAI021775 | Palldipes | GpaPLP10a | Peritrophin-Like Protein | Chitin-binding, Peritrophin-A-Domain, mucin domains |
|  | GPAI021777 | Palldipes | GpaPLP10b | Peritrophin-Like Protein | Chitin-binding, Peritrophin-A-Domain, mucin domains |
|  | GPPI024565 | Palpalis | GppPLP10 | Peritrophin-Like Protein | Chitin-binding, Peritrophin-A-Domain, mucin domains |
|  |  |  |  |  |  |
| FBgn0260386 | GMOY002812 | Morsitans | GmmPLP11 | Peritrophin-Like Protein | Chitin-binding, Peritrophin-A-Domain |
|  | GAUT028770 | Austeni | GauPLP11 | Peritrophin-Like Protein | Chitin-binding, Peritrophin-A-Domain |
|  | GBRI019411 | Brevipalpis | GbrPLP11 | Peritrophin-Like Protein | Chitin-binding, Peritrophin-A-Domain |
|  | GFUI040609 | Fuscipes | GfuPLP11 | Peritrophin-Like Protein | Chitin-binding, Peritrophin-A-Domain |
|  | GPAI030232 | Palldipes | GpaPLP11 | Peritrophin-Like Protein | Chitin-binding, Peritrophin-A-Domain |
|  | GPPI040873 | Palpalis | GppPLP11 | Peritrophin-Like Protein | Chitin-binding, Peritrophin-A-Domain |
|  |  |  |  |  |  |
| FBgn0031737 | GMOY003840 | Morsitans | GmmPLP12 | Peritrophin-Like Protein | Chitin-binding, Peritrophin-A-Domain |
|  | GAUT040598 | Austeni | GauPLP12 | Peritrophin-Like Protein | Chitin-binding, Peritrophin-A-Domain |
|  | GBRI039371 | Brevipalpis | GbrPLP12 | Peritrophin-Like Protein | Chitin-binding, Peritrophin-A-Domain |
|  | GFUI049678 | Fuscipes | GfuPLP12 | Peritrophin-Like Protein | Chitin-binding, Peritrophin-A-Domain |
|  | GPAI033642 | Palldipes | GpaPLP12 | Peritrophin-Like Protein | Chitin-binding, Peritrophin-A-Domain |
|  | GPPI003309 | Palpalis | GppPLP12 | Peritrophin-Like Protein | Chitin-binding, Peritrophin-A-Domain |
|  |  |  |  |  |  |
|  | GMOY004823 | Morsitans | GmmPLP13 | Peritrophin-Like Protein | Chitin-binding, Peritrophin-A-Domain |
|  | GAUT015903 | Austeni | GauPLP13 | Peritrophin-Like Protein | Chitin-binding, Peritrophin-A-Domain |
|  | GFUI012030 | Fuscipes | GfuPLP13a | Peritrophin-Like Protein | Chitin-binding, Peritrophin-A-Domain |
|  | GFUI017945 | Fuscipes | GfuPLP13b | Peritrophin-Like Protein | Chitin-binding, Peritrophin-A-Domain |
|  | GPAI009564 | Palldipes | GpaPLP13 | Peritrophin-Like Protein | Chitin-binding, Peritrophin-A-Domain |
|  |  |  |  |  |  |
| FBgn0027600 | GMOY004893 | Morsitans | GmmPLP14 | Peritrophin-Like Protein | Chitin-binding, Peritrophin-A-Domain |
|  | GAUT011300 | Austeni | GauPLP14 | Peritrophin-Like Protein | Chitin-binding, Peritrophin-A-Domain |
|  | GBRI022913 | Brevipalpis | GbrPLP14 | Peritrophin-Like Protein | Chitin-binding, Peritrophin-A-Domain |
|  | GFUI015986 | Fuscipes | GfuPLP14 | Peritrophin-Like Protein | Chitin-binding, Peritrophin-A-Domain |
|  | GPAI048004 | Palldipes | GpaPLP14 | Peritrophin-Like Protein | Chitin-binding, Peritrophin-A-Domain |
|  | GPPI007754 | Palpalis | GppPLP14 | Peritrophin-Like Protein | Chitin-binding, Peritrophin-A-Domain |
|  |  |  |  |  |  |
| FBgn0025390 | GMOY005251 | Morsitans | GmmPLP15 | Peritrophin-Like Protein | Chitin-binding, Peritrophin-A-Domain |
|  | GBRIO26889 | Brevipalpis | GbrPLP15 | Peritrophin-Like Protein | Chitin-binding, Peritrophin-A-Domain |
|  | GFUI045474 | Fuscipes | GfuPLP15a | Peritrophin-Like Protein | Chitin-binding, Peritrophin-A-Domain |
|  | GFUI050311 | Fuscipes | GfuPLP15b | Peritrophin-Like Protein | Chitin-binding, Peritrophin-A-Domain |
|  | GPAI039542 | Palldipes | GpaPLP15 | Peritrophin-Like Protein | Chitin-binding, Peritrophin-A-Domain |
|  | GPPI018985 | Palpalis | GppPLP15 | Peritrophin-Like Protein | Chitin-binding, Peritrophin-A-Domain |
|  |  |  |  |  |  |
| FBgn0038422 | GMOY005235 | Morsitans | GmmPLP16 | Peritrophin-Like Protein | Chitin-binding, Peritrophin-A-Domain |
|  | GAUT010029 | Austeni | GauPLP16 | Peritrophin-Like Protein | Chitin-binding, Peritrophin-A-Domain |
|  | GBRI008201 | Brevipalpis | GbrPLP16 | Peritrophin-Like Protein | Chitin-binding, Peritrophin-A-Domain |
|  | GFUI023663 | Fuscipes | GfuPLP16 | Peritrophin-Like Protein | Chitin-binding, Peritrophin-A-Domain |
|  | GPAI033432 | Palldipes | GpaPLP16 | Peritrophin-Like Protein | Chitin-binding, Peritrophin-A-Domain |
|  | GPPI023739 | Palpalis | GppPLP16 | Peritrophin-Like Protein | Chitin-binding, Peritrophin-A-Domain |
|  |  |  |  |  |  |
| FBgn0038492 | GMOY005278 | Morsitans | GmmPLP17 | Peritrophin-Like Protein | Chitin-binding, Peritrophin-A-Domain |
|  | GAUT029524 | Austeni | GauPLP17a | Peritrophin-Like Protein | Chitin-binding, Peritrophin-A-Domain |
|  | GAUT029525 | Austeni | GauPLP17b | Peritrophin-Like Protein | Chitin-binding, Peritrophin-A-Domain |
|  | GBRI026281 | Brevipalpis | GbrPLP17 | Peritrophin-Like Protein | Chitin-binding, Peritrophin-A-Domain |
|  | GFUI015251 | Fuscipes | GfuPLP17 | Peritrophin-Like Protein | Chitin-binding, Peritrophin-A-Domain |
|  | GPAI036992 | Palldipes | GpaPLP17 | Peritrophin-Like Protein | Chitin-binding, Peritrophin-A-Domain |
|  | GPPI048759 | Palpalis | GppPLP17a | Peritrophin-Like Protein | Chitin-binding, Peritrophin-A-Domain |
|  | GPPI042690 | Palpalis | GppPLP17b | Peritrophin-Like Protein | Chitin-binding, Peritrophin-A-Domain |
|  |  |  |  |  |  |
| FBgn0034030 | GMOY006713 | Morsitans | GmmPLP18 | Peritrophin-Like Protein | Chitin-binding, Peritrophin-A-Domain |
|  | GAUT023971 | Austeni | GauPLP18 | Peritrophin-Like Protein | Chitin-binding, Peritrophin-A-Domain |
|  | GFUI042924 | Fuscipes | GfuPLP18 | Peritrophin-Like Protein | Chitin-binding, Peritrophin-A-Domain |
|  | GPAI039717 | Palldipes | GpaPLP18 | Peritrophin-Like Protein | Chitin-binding, Peritrophin-A-Domain |
|  | GPPI013212 | Palpalis | GppPLP18 | Peritrophin-Like Protein | Chitin-binding, Peritrophin-A-Domain |
|  |  |  |  |  |  |
| FBgn0036203 | GMOY007191 | Morsitans | GmmPLP19  GmmPer108 | Peritrophin-Like Protein | Chitin-binding, Peritrophin-A-Domain |
|  | GAUT013406 | Austeni | GauPLP19 | Peritrophin-Like Protein | Chitin-binding, Peritrophin-A-Domain |
|  | GBRI016187 | Brevipalpis | GbrPLP19 | Peritrophin-Like Protein | Chitin-binding, Peritrophin-A-Domain |
|  | GFUI029181 | Fuscipes | GfuPLP19 | Peritrophin-Like Protein | Chitin-binding, Peritrophin-A-Domain |
|  | GPAI035996 | Palldipes | GpaPLP19 | Peritrophin-Like Protein | Chitin-binding, Peritrophin-A-Domain |
|  | GPPI010524 | Palpalis | GppPLP19 | Peritrophin-Like Protein | Chitin-binding, Peritrophin-A-Domain |
|  |  |  |  |  |  |
| FBgn0040601 | GMOY007476 | Morsitans | GmmPLP20 | Peritrophin-Like Protein | Chitin-binding, Partial Peritrophin-C-Domain |
|  | GAUT039321 | Austeni | GauPLP20 | Peritrophin-Like Protein | Chitin-binding, Partial Peritrophin-C-Domain |
|  | GBRI029781 | Brevipalpis | GbrPLP20 | Peritrophin-Like Protein | Chitin-binding, Partial Peritrophin-C-Domain |
|  | GFUI006871 | Fuscipes | GfuPLP20 | Peritrophin-Like Protein | Chitin-binding, Partial Peritrophin-C-Domain |
|  | GPAI013818 | Palldipes | GpaPLP20 | Peritrophin-Like Protein | Chitin-binding, Partial Peritrophin-C-Domain |
|  |  |  |  |  |  |
| FBgn0036845 | GMOY008030 | Morsitans | GmmPLP21 | Peritrophin-Like Protein | Chitin-binding, Peritrophin-A-Domain |
|  | GAUT042200 | Austeni | GauPLP21 | Peritrophin-Like Protein | Chitin-binding, Peritrophin-A-Domain |
|  | GBRI039692 | Brevipalpis | GbrPLP21 | Peritrophin-Like Protein | Chitin-binding, Peritrophin-A-Domain |
|  | GFUI011847 | Fuscipes | GfuPLP21 | Peritrophin-Like Protein | Chitin-binding, Peritrophin-A-Domain |
|  | GPAI019290 | Palldipes | GpaPLP21 | Peritrophin-Like Protein | Chitin-binding, Peritrophin-A-Domain |
|  | GPPI004759 | Palpalis | GppPLP21 | Peritrophin-Like Protein | Chitin-binding, Peritrophin-A-Domain |
|  |  |  |  |  |  |
| FBgn0035844 | GMOY008032 | Morsitans | GmmPLP22 | Peritrophin-Like Protein | Chitin-binding, Peritrophin-A-Domain |
|  | GAUT042214 | Austeni | GauPLP22 | Peritrophin-Like Protein | Chitin-binding, Peritrophin-A-Domain |
|  | GBRI039674 | Brevipalpis | GbrPLP22 | Peritrophin-Like Protein | Chitin-binding, Peritrophin-A-Domain |
|  | GFUI011826 | Fuscipes | GfuPLP22 | Peritrophin-Like Protein | Chitin-binding, Peritrophin-A-Domain |
|  | GPAI019295 | Palldipes | GpaPLP22 | Peritrophin-Like Protein | Chitin-binding, Peritrophin-A-Domain |
|  | GPPI004758 | Palpalis | GppPLP22 | Peritrophin-Like Protein | Chitin-binding, Peritrophin-A-Domain |
|  |  |  |  |  |  |
| FBgn0040959 | GMOY009587 | Morsitans | GmmPLP23/Pro2 | Peritrophin-Like Protein | Chitin-binding, Partial Peritrophin-C-Domain |
|  | GAUT008830 | Austeni | GauPLP23a | Peritrophin-Like Protein | Chitin-binding, Partial Peritrophin-C-Domain |
|  | GAUT024681 | Austeni | GauPLP23b | Peritrophin-Like Protein | Chitin-binding, Partial Peritrophin-C-Domain |
|  | GBRI017781 | Brevipalpis | GbrPLP23 | Peritrophin-Like Protein | Chitin-binding, Partial Peritrophin-C-Domain |
|  | GFUI019092 | Fuscipes | GfuPLP23 | Peritrophin-Like Protein | Chitin-binding, Partial Peritrophin-C-Domain |
|  | GPAI002755 | Palldipes | GpaPLP23 | Peritrophin-Like Protein | Chitin-binding, Partial Peritrophin-C-Domain |
|  | GPPI039564 | Palpalis | GppPLP23a | Peritrophin-Like Protein | Chitin-binding, Partial Peritrophin-C-Domain |
|  | GPPI022981 | Palpalis | GppPLP23b | Peritrophin-Like Protein | Chitin-binding, Partial Peritrophin-C-Domain |
|  | GPPI039559 | Palpalis | GppPLP23c | Peritrophin-Like Protein | Chitin-binding, Partial Peritrophin-C-Domain |
|  |  |  |  |  |  |
| FBgn0036230 | GMOY009788 | Morsitans | GmmPLP24 | Peritrophin-Like Protein | Chitin-binding, Peritrophin-A-Domain |
| FBgn0262986 | GAUT012373 | Austeni | GauPLP24 | Peritrophin-Like Protein | Chitin-binding, Peritrophin-A-Domain |
|  | GFUI027349 | Brevipalpis | GbrPLP24 | Peritrophin-Like Protein | Chitin-binding, Peritrophin-A-Domain |
|  | GPAI002186 | Palldipes | GpaPLP24 | Peritrophin-Like Protein | Chitin-binding, Peritrophin-A-Domain |
|  | GPPI017724 | Palpalis | GppPLP24 | Peritrophin-Like Protein | Chitin-binding, Peritrophin-A-Domain |
|  |  |  |  |  |  |
| FBgn0036230 | GMOY009805 | Morsitans | GmmPLP25 | Peritrophin-Like Protein | Chitin-binding, Peritrophin-A-Domain |
| FBgn0262986 | GAUT012335 | Austeni | GauPLP25 | Peritrophin-Like Protein | Chitin-binding, Peritrophin-A-Domain |
|  | GFUI026256 | Fuscipes | GfuPLP25 | Peritrophin-Like Protein | Chitin-binding, Peritrophin-A-Domain |
|  | GPAI002122 | Palldipes | GpaPLP25a | Peritrophin-Like Protein | Chitin-binding, Peritrophin-A-Domain |
|  | GPAI002124 | Palldipes | GpaPLP25b | Peritrophin-Like Protein | Chitin-binding, Peritrophin-A-Domain |
|  | GPPI016003 | Palpalis | GppPLP25 | Peritrophin-Like Protein | Chitin-binding, Peritrophin-A-Domain |
|  |  |  |  |  |  |
| FBgn0036226 | GMOY009806 | Morsitans | GmmPLP26a | Peritrophin-Like Protein | Chitin-binding, Peritrophin-A-Domain |
|  | GMOY009807 | Morsitans | GmmPLP26b | Peritrophin-Like Protein | Chitin-binding, Peritrophin-A-Domain |
|  | GAUT012312 | Austeni | GauPLP26a | Peritrophin-Like Protein | Chitin-binding, Peritrophin-A-Domain |
|  | GAUT012316 | Austeni | GauPLP26b | Peritrophin-Like Protein | Chitin-binding, Peritrophin-A-Domain |
|  | GBRI029501 | Brevipalpis | GbrPLP26 | Peritrophin-Like Protein | Chitin-binding, Peritrophin-A-Domain |
|  | GFUI026249 | Fuscipes | GfuPLP26a | Peritrophin-Like Protein | Chitin-binding, Peritrophin-A-Domain |
|  | GFUI026252 | Fuscipes | GfuPLP26b | Peritrophin-Like Protein | Chitin-binding, Peritrophin-A-Domain |
|  | GPAI002108 | Palldipes | GpaPLP26 | Peritrophin-Like Protein | Chitin-binding, Peritrophin-A-Domain |
|  | GPPI016000 | Palpalis | GppPLP26 | Peritrophin-Like Protein | Chitin-binding, Peritrophin-A-Domain |
|  |  |  |  |  |  |
| FBgn0022770 | GMOY009571 | Morsitans | GmmPLP27 | Peritrophin-Like Protein | Chitin-binding, Peritrophin-A-Domain |
|  | GAUT032039 | Austeni | GauPLP27 | Peritrophin-Like Protein | Chitin-binding, Peritrophin-A-Domain |
|  | GFUI025256 | Fuscipes | GfuPLP27 | Peritrophin-Like Protein | Chitin-binding, Peritrophin-A-Domain |
|  | GPAI004770 | Palldipes | GpaPLP27 | Peritrophin-Like Protein | Chitin-binding, Peritrophin-A-Domain |
|  | GPPI004131 | Palpalis | GppPLP27 | Peritrophin-Like Protein | Chitin-binding, Peritrophin-A-Domain |
|  |  |  |  |  |  |
| FBgn0031097 | GMOY009572 | Morsitans | GmmPLP28 | Peritrophin-Like Protein | Chitin-binding, Peritrophin-A-Domain |
|  | GAUT032040 | Austeni | GauPLP28 | Peritrophin-Like Protein | Chitin-binding, Peritrophin-A-Domain |
|  | GBRI003555 | Brevipalpis | GbrPLP28 | Peritrophin-Like Protein | Chitin-binding, Peritrophin-A-Domain |
|  | GFUI025254 | Fuscipes | GfuPLP28 | Peritrophin-Like Protein | Chitin-binding, Peritrophin-A-Domain |
|  | GPAI004766 | Palldipes | GpaPLP28 | Peritrophin-Like Protein | Chitin-binding, Peritrophin-A-Domain |
|  | GPPI004133 | Palpalis | GppPLP28 | Peritrophin-Like Protein | Chitin-binding, Peritrophin-A-Domain |
|  |  |  |  |  |  |
| FBgn0026077 | GMOY009826 | Morsitans | GmmPLP29 | Peritrophin-Like Protein | Chitin-binding, Peritrophin-A-Domain |
|  | GAUT041130 | Austeni | GauPLP29 | Peritrophin-Like Protein | Chitin-binding, Peritrophin-A-Domain |
|  | GBRI031719 | Brevipalpis | GbrPLP29 | Peritrophin-Like Protein | Chitin-binding, Peritrophin-A-Domain |
|  | GFUI037199 | Fuscipes | GfuPLP29a | Peritrophin-Like Protein | Chitin-binding, Peritrophin-A-Domain |
|  | GFUI049166 | Fuscipes | GfuPLP29b | Peritrophin-Like Protein | Chitin-binding, Peritrophin-A-Domain |
|  | GPA1031701 | Palldipes | GpaPLP29 | Peritrophin-Like Protein | Chitin-binding, Peritrophin-A-Domain |
|  |  |  |  |  |  |
|  | GMOY011054 | Morsitans | GmmPLP30 | Peritrophin-Like Protein | Chitin-binding, Peritrophin-A-Domain |
|  | GAUT026340 | Austeni | GauPLP30 | Peritrophin-Like Protein | Chitin-binding, Peritrophin-A-Domain |
|  | GBRI013108 | Brevipalpis | GbrPLP30 | Peritrophin-Like Protein | Chitin-binding, Peritrophin-A-Domain |
|  | GFUI045125 | Fuscipes | GfuPLP30 | Peritrophin-Like Protein | Chitin-binding, Peritrophin-A-Domain |
|  | GPAI027171 | Palldipes | GpaPLP30 | Peritrophin-Like Protein | Chitin-binding, Peritrophin-A-Domain |
|  | GPPI005116 | Palpalis | GppPLP30 | Peritrophin-Like Protein | Chitin-binding, Peritrophin-A-Domain |
|  |  |  |  |  |  |
| FBgn0039452 | GMOY011777 | Morsitans | GmmPLP31 | Peritrophin-Like Protein | Chitin-binding, Partial Peritrophin-C-Domain |
|  | GAUT036980 | Austeni | GauPLP31a | Peritrophin-Like Protein | Chitin-binding, Partial Peritrophin-C-Domain |
|  | GAUT039327 | Austeni | GauPLP31b | Peritrophin-Like Protein | Chitin-binding, Partial Peritrophin-C-Domain |
|  | GBRI013425 | Brevipalpis | GbrPLP31a | Peritrophin-Like Protein | Chitin-binding, Partial Peritrophin-C-Domain |
|  | GBRI034385 | Brevipalpis | GbrPLP31b | Peritrophin-Like Protein | Chitin-binding, Partial Peritrophin-C-Domain |
|  | GFUI006839 | Fuscipes | GfuPLP31a | Peritrophin-Like Protein | Chitin-binding, Partial Peritrophin-C-Domain |
|  | GFUI006847 | Fuscipes | GfuPLP31b | Peritrophin-Like Protein | Chitin-binding, Partial Peritrophin-C-Domain |
|  | GPAI013834 | Palldipes | GpaPLP31a | Peritrophin-Like Protein | Chitin-binding, Partial Peritrophin-C-Domain |
|  | GPAI013840 | Palldipes | GpaPLP31b | Peritrophin-Like Protein | Chitin-binding, Partial Peritrophin-C-Domain |
|  | GPPI050938 | Palpalis | GppPLP31a | Peritrophin-Like Protein | Chitin-binding, Partial Peritrophin-C-Domain |
|  | GPPI050939 | Palpalis | GppPLP31b | Peritrophin-Like Protein | Chitin-binding, Partial Peritrophin-C-Domain |
|  |  |  |  |  |  |
| FBgn0038643 | GMOY011809 | Morsitans | GmmPLP32/Pro1 | Peritrophin-Like Protein | Chitin-binding, Partial Peritrophin-C-Domain |
|  | GBRI007305 | Brevipalpis | GbrPLP32 | Peritrophin-Like Protein | Chitin-binding, Partial Peritrophin-C-Domain |
|  | GPPI040997 | Palpalis | GpaPLP32 | Peritrophin-Like Protein | Chitin-binding, Partial Peritrophin-C-Domain |
|  |  |  |  |  |  |
| FBgn0085311 | GMOY011810 | Morsitans | GmmPLP33  GmmPer12 | Peritrophin-Like Protein | Chitin-binding, Partial Peritrophin-C-Domain |
|  | GAUT038910 | Austeni | GauPLP33 | Peritrophin-Like Protein | Chitin-binding, Partial Peritrophin-C-Domain |
|  | GBRI007300 | Brevipalpis | GbrPLP33a | Peritrophin-Like Protein | Chitin-binding, Partial Peritrophin-C-Domain |
|  | GBRI007301 | Brevipalpis | GbrPLP33b | Peritrophin-Like Protein | Chitin-binding, Partial Peritrophin-C-Domain |
|  | GFUI000259 | Fuscipes | GfuPLP33 | Peritrophin-Like Protein | Chitin-binding, Partial Peritrophin-C-Domain |
|  | GPPI040977 | Palpalis | GppPLP33 | Peritrophin-Like Protein | Chitin-binding, Partial Peritrophin-C-Domain |
|  |  |  |  |  |  |
|  | GAUT012314 | Austeni | GauPLP34 | Peritrophin-Like Protein | Chitin-binding, Peritrophin-A-Domain, mucin domains |
|  | GBRI029506 | Brevipalpis | GbrPLP34 | Peritrophin-Like Protein | Chitin-binding, Peritrophin-A-Domain, mucin domains |
|  | GFUI026251 | Fuscipes | GfuPLP34 | Peritrophin-Like Protein | Chitin-binding, Peritrophin-A-Domain, mucin domains |
|  | GPAI002117 | Palldipes | GpaPLP34 | Peritrophin-Like Protein | Chitin-binding, Peritrophin-A-Domain, mucin domains |
|  | GPPI016008 | Palpalis | GppPLP34 | Peritrophin-Like Protein | Chitin-binding, Peritrophin-A-Domain, mucin domains |
|  |  |  |  |  |  |
|  | GAUT044451 | Austeni | GauPLP35 | Peritrophin-Like Protein | Chitin-binding, Peritrophin-A-Domain |
|  | GBRI023665 | Brevipalpis | GbrPLP35 | Peritrophin-Like Protein | Chitin-binding, Peritrophin-A-Domain |
|  | GFUI048633 | Fuscipes | GfuPLP35 | Peritrophin-Like Protein | Chitin-binding, Peritrophin-A-Domain |
|  | GPPI048544 | Palpalis | GppPLP35 | Peritrophin-Like Protein | Chitin-binding, Peritrophin-A-Domain |
|  |  |  |  |  |  |
|  | GAUT036465 | Austeni | GauPLP36 | Peritrophin-Like Protein | Chitin-binding, Partial Peritrophin-C-Domain |
|  | GBRI019611 | Brevipalpis | GbrPLP36 | Peritrophin-Like Protein | Chitin-binding, Partial Peritrophin-C-Domain |
|  | GPAI033924 | Palldipes | GppPLP36 | Peritrophin-Like Protein | Chitin-binding, Partial Peritrophin-C-Domain |
|  |  |  |  |  |  |
| FBgn0040958 | GAUT051817 | Austeni | GauPLP37 | Peritrophin-Like Protein | Chitin-binding, Partial Peritrophin-C-Domain |
|  | GBRI017597 | Brevipalpis | GbrPLP37 | Peritrophin-Like Protein | Chitin-binding, Partial Peritrophin-C-Domain |
|  | GFUI036185 | Fuscipes | GfuPLP37 | Peritrophin-Like Protein | Chitin-binding, Partial Peritrophin-C-Domain |
|  |  |  |  |  |  |
| FBgn0259192 | GBRI023004 | Brevipalpis | GbrPLP38 | Peritrophin-Like Protein | Chitin-binding, Peritrophin-A-Domain |
|  | GFUI007992 | Fuscipes | GfuPLP38 | Peritrophin-Like Protein | Chitin-binding, Peritrophin-A-Domain |
|  | GPAI006518 | Palldipes | GpaPLP38 | Peritrophin-Like Protein | Chitin-binding, Peritrophin-A-Domain |
|  | GPPI046699 | Palpalis | GppPLP38 | Peritrophin-Like Protein | Chitin-binding, Peritrophin-A-Domain |
|  |  |  |  |  |  |
|  | GBRI024198 | Brevipalpis | GbrPLP39 | Peritrophin-Like Protein | Chitin-binding, Peritrophin-A-Domain |
|  | GFUI022163 | Fuscipes | GfuPLP39 | Peritrophin-Like Protein | Chitin-binding, Peritrophin-A-Domain |
|  | GPAI033201 | Palldipes | GpaPLP39 | Peritrophin-Like Protein | Chitin-binding, Peritrophin-A-Domain |
|  | GPPI000357 | Palpalis | GppPLP39 | Peritrophin-Like Protein | Chitin-binding, Peritrophin-A-Domain |
|  |  |  |  |  |  |
|  | GBRI027291 | Brevipalpis | GbrPLP40 | Peritrophin-Like Protein | Chitin-binding, Peritrophin-A-Domain |
|  | GPAI016225 | Palldipes | GpaPLP40 | Peritrophin-Like Protein | Chitin-binding, Peritrophin-A-Domain |
|  |  |  |  |  |  |
|  | GFUI037693 | Fuscipes | GfuPLP41 | Peritrophin-Like Protein | Chitin-binding, Peritrophin-A-Domain |
|  | GPPI030394 | Palpalis | GppPLP41 | Peritrophin-Like Protein | Chitin-binding, Peritrophin-A-Domain |
|  |  |  |  |  |  |
|  | GFUI022165 | Fuscipes | GfuPLP42 | Peritrophin-Like Protein | Chitin-binding, Peritrophin-A-Domain |

**Supplemental Table 15:** Details of the primers used to amplify the mitochondrial genome of the seven-tsetse species and for HRM analysis.

| Primers to amplify tsetse species mtDNA genome | |
| --- | --- |
| Primer pair | Primer sequence (5’🡺3’) |
| MtDNAtseF1 | TGATAAAWTTTGTGCCAGCAATCG |
| MtDNAtseR1 | TCCAGTACAYCTACTATGTTACGACT |
| MtDNAtseF2 | ATGCACATATCGCCCGTCRCTCTT |
| MtDNAtseR2 | ATAGAAACCAACCTGGCTYACR |
| MtDNAtseF3 | AGCCAGGTTGGTTTCTATCTTT |
| MtDNAtseR3 | TTAACHTGAATTGGAGCYCGACCW |
| MtDNAtseF4 | WGGTCGRGCTCCAATTCADGTT |
| MtDNAtseR4 | TTAGTHCAATGAGTWTGAGGWGGH |
| MtDNAtseF5 | DCCWCCTCAWACTCATTGDACTA |
| MtDNAtseR5 | AATCATTHCCATGWGTDCGAATW |
| MtDNAtseF6 | WATTCGHACWCATGGDAATGAT |
| MtDNAtseR6 | CAGGRGCTTCTACATGAGCTTTAGG |
| MtDNAtseF7 | TAAAGCTCATGTAGAAGCYCCTGT |
| MtDNAtseR7 | TCAAAYTCATARTTAGCCCCTAAHCCN |
| MtDNAtseF8 | NGGDTTAGGGGCTAAYTATGARTTTGA |
| MtDNAtseR8 | CAATCTWCCTTGAATATGAAGCG |
| MtDNAtseF9 | GCAGCTTTTWCTTGRACTTCATACTT |
| MtDNAtseR9 | TCAGGDATTACTGTDACTTGAGCY |
| MtDNAtseF10 | GCTCAAGTHACAGTAATHCCTGADGT |
| MtDNAtseR10 | AGATGACTGAAAGCAAGTAYTGGTC |
| MtDNAtseF11 | GACCARTACTTGCTTTCAGTCATC |
| MtDNAtseR11 | YTAGCNGGWATACCTCGTCGY |
| MtDNAtseF12 | RCGACGAGGTATWCCNGCTAR |
| MtDNAtseR12 | TTCAGCCATTTAATCGCRACARTGA |
| MtDNAtseF13 | YGCGATTAAATGGCTGAAKW |
| MtDNAtseR13 | GCTAAHHAAGCTAMTGGGTTCATACC |
| MtDNAtseF14 | RATGGGGTATGAACCCAKTAGC |
| MtDNAtseR14 | CCTCTRAATAGACTAAAATACCGCCA |
| Primers for HRM analysis of tsetse populations/haplotypes | |
| TsetseHRM-F | TAGCCCCTAACCCCGCTATAAAT |
| TsetseHRM-R | TTCCATTTTCTTCTTGATTGCCTGC |

**Supplemental Table 16: SRA Numbers and Associated Information on *Glossina* RNA-seq Datasets**

| Accession | Sample Name | Link to Datafile | Organism | Tax ID |
| --- | --- | --- | --- | --- |
| SRR7698168 | Whole Male *G. brevipalpis* RNA-seq | https://www.ncbi.nlm.nih.gov/Traces/sra/?run=SRR7698168 | *Glossina brevipalpis* | 37001 |
| SRR7698169 | Non-lactating Whole Female *G. brevipalpis* RNA-seq | <https://www.ncbi.nlm.nih.gov/Traces/sra/?run=SRR7698169> | *Glossina brevipalpis* | 37001 |
| SRR7698166 | Lactating Whole Female *G. brevipalpis* RNA-seq | <https://www.ncbi.nlm.nih.gov/Traces/sra/?run=SRR7698166> | *Glossina brevipalpis* | 37001 |
| SRR7698167 | Whole Male *G. fuscipes* RNA-seq | <https://www.ncbi.nlm.nih.gov/Traces/sra/?run=SRR7698167> | *Glossina fuscipes* | 7396 |
| SRR7698164 | Non-lactating Whole Female *G. fuscipes* RNA-seq | <https://www.ncbi.nlm.nih.gov/Traces/sra/?run=SRR7698164> | *Glossina fuscipes* | 7396 |
| SRR7698165 | Lactating Whole Female *G. fuscipes* RNA-seq | <https://www.ncbi.nlm.nih.gov/Traces/sra/?run=SRR7698165> | *Glossina fuscipes* | 7396 |
| SRR7698162 | Whole Male *G. pallidipes* RNA-seq | <https://www.ncbi.nlm.nih.gov/Traces/sra/?run=SRR7698162> | *Glossina pallidipes* | 7398 |
| SRR7698163 | Non-lactating Whole Female *G. pallidipes* RNA-seq | <https://www.ncbi.nlm.nih.gov/Traces/sra/?run=SRR7698163> | *Glossina pallidipes* | 7398 |
| SRR7698160 | Lactating Whole Female *G. pallidipes* RNA-seq | <https://www.ncbi.nlm.nih.gov/Traces/sra/?run=SRR7698160> | *Glossina pallidipes* | 7398 |
| SRR7698161 | Whole Male *G. palpalis gambiensis* RNA-seq | <https://www.ncbi.nlm.nih.gov/Traces/sra/?run=SRR7698161> | *Glossina palpalis gambiensis* | 67801 |
| SRR7698158 | Non-lactating Whole Female *G. palpalis* *gambiensis* RNA-seq | <https://www.ncbi.nlm.nih.gov/Traces/sra/?run=SRR7698158> | *Glossina palpalis gambiensis* | 67801 |
| SRR7698159 | Lactating Whole Female *G. palpalis gambiensis* RNA-seq | <https://www.ncbi.nlm.nih.gov/Traces/sra/?run=SRR7698159> | *Glossina palpalis gambiensis* | 67801 |
| SRS2364381 | Male Reproductive tract *G. morsitans* RNA-seq | <https://www.ncbi.nlm.nih.gov/sra/SRS2364381> | *Glossina morsitans morsitans* | 37546 |
| SRS430099 | Non-lactating Whole Female *G. morsitans* RNA-seq | <https://www.ncbi.nlm.nih.gov/sra/SRS430099> | *Glossina morsitans morsitans* | 37546 |
| SRS430097 | Lactating Whole Female *G. morsitans* RNA-seq | <https://www.ncbi.nlm.nih.gov/sra/SRS430097> | *Glossina morsitans morsitans* | 37546 |
| SRS686445 | Whole Male *G. austeni* RNA-seq | <https://www.ncbi.nlm.nih.gov/sra/SRX682955> | *Glossina austeni* | 7395 |
| SRS686473 | Non-lactating Whole Female *G. austeni* RNA-seq | <https://www.ncbi.nlm.nih.gov/sra/SRX682983> | *Glossina austeni* | 7395 |
