# Supplementary material for: Comparative genomic analysis of six Glossina genomes, vectors of African trypanosomes
Source: Genome Biol. 2019 Sep 2;20:187. doi: 10.1186/s13059-019-1768-2 (PMC6721284; doi:10.1186/s13059-019-1768-2)

Introduction - Supplemental Figure Legends

Supplemental Figure 1: Total genomic coverage of Transposable Element clusters across the Glossina genomes in order of abundance and colored by conservation across species. A. Conservation of LINE/CR1 type clusters across the genomes of the six tsetse species. B. Conservation of the DNA/mariner type clusters across the genomes of the six tsetse species. Abbreviations: G. aus (*Glossina austeni*), G. bre (*Glossina brevipalpis*), G. fus (*Glossina fuscipes*), G. mor (*Glossina morsitans*), G pal (*Glossina pallidipes*), G. pap (*Glossina palpalis*)

Supplemental Figure 2: Maximum Likelihood, Bayesian and Astral based phylogenetic analysis of a concatenated single gene ortholog alignment. A: Maximum likelihood tree inferred by Raxml on a concatenated alignment of 117,783 amino acids from 286 genes and employing the LG+G+F model of replacement. Value at nodes are bootstrap supports from the consensus of 100 pseudoreplicates in Raxml. B: Bayesian phylogenic tree inferred with Phylobayes 3 using CAT+G replacment model, supports at nodes are posterior probabilities; C: Astral analysis based on 286 genes with bootstrap support.

Supplemental Figure 3: Tsetse variable mitochondrial DNA sequence and species delineation by high resolution melt curve analysis

Supplemental Figure 4: Application of high-resolution melt curve analysis to distinguish Tsetse haplotypes/populations

Supplemental Figure 5: The percent of female-, male-, and un-biased genes that are on an X chromosome scaffold (Muller elements A, D, or F) is plotted for each species. Sex-biased expression was measured between males and either lactating (L) or non-lactating (NL) females. Asterisks indicate a significant difference between the percent of sex-biased genes that are X-linked when compared to unbiased genes (*$P<0.05$ in Fisher's exact test).

Supplemental Figure 6: The distribution of the log2 (fold-change between females and males) is plotted for autosomal and X-linked genes in each species. Female-male gene expression comparisons are between males and either lactating or non-lactating females.

Supplemental Figure 7: Rates of non-synonymous to synonymous substitution (dN/dS) along different evolutionary lineages within the *Glossina* genus.

Supplemental Figure 8: Rates of non-synonymous to synonymous substitution (dN/dS) rates of female, male and non-sex biased genes across the X and autosomal muller elements.

Supplemental Figure 9: The distribution of male biased gene expression across the predicted *Glossina* Muller Elements. Bar heights represent the percentage of genes per element with male biased gene expression.

Supplemental Figure 10: Rates of non-synonymous to synonymous substitution (dN/dS) rates across the predicted muller elements.

Supplemental Figure 11: Sub-genus specific gene family expansions/retractions (with orthology group number annotations). Principal component analysis-based clustering of gene orthology groups showing significant differences in the number of representative sequences between the six *Glossina* species.

Supplemental Figure 12: Distribution of transcription factor families across insect genomes. Heatmap depicting the abundance of transcription factor (TF) families across a collection of insect genomes. Each entry indicates the number of TF genes for the given family in the given genome, based on presence of DNA binding domains. Color key is depicted at the top (light blue means the TF family is completely absent) – note log (base 2) scale.

Suppplemental Figure 1 : Total genomic coverage of TE clusters across the Glossina genomes in order of abundance and colored by conservation across species

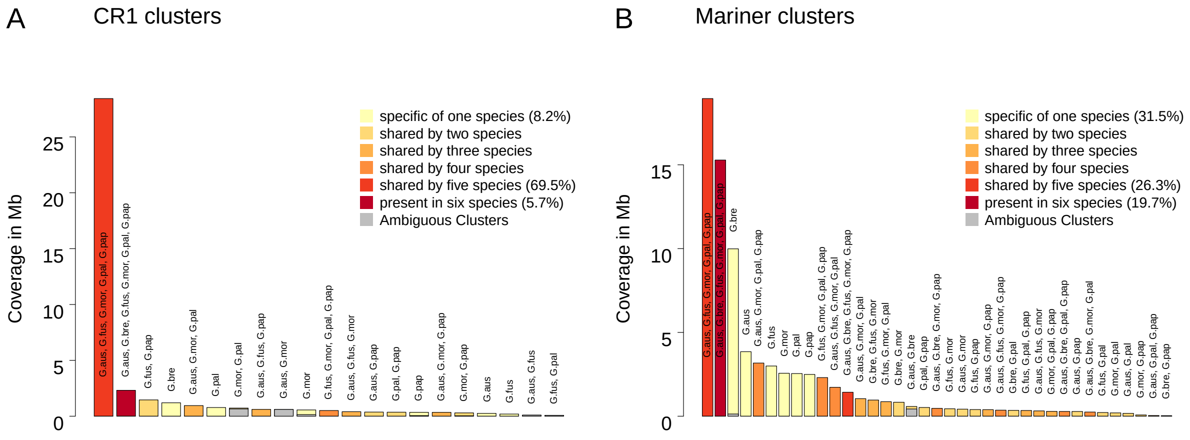

Supplemental Figure 2

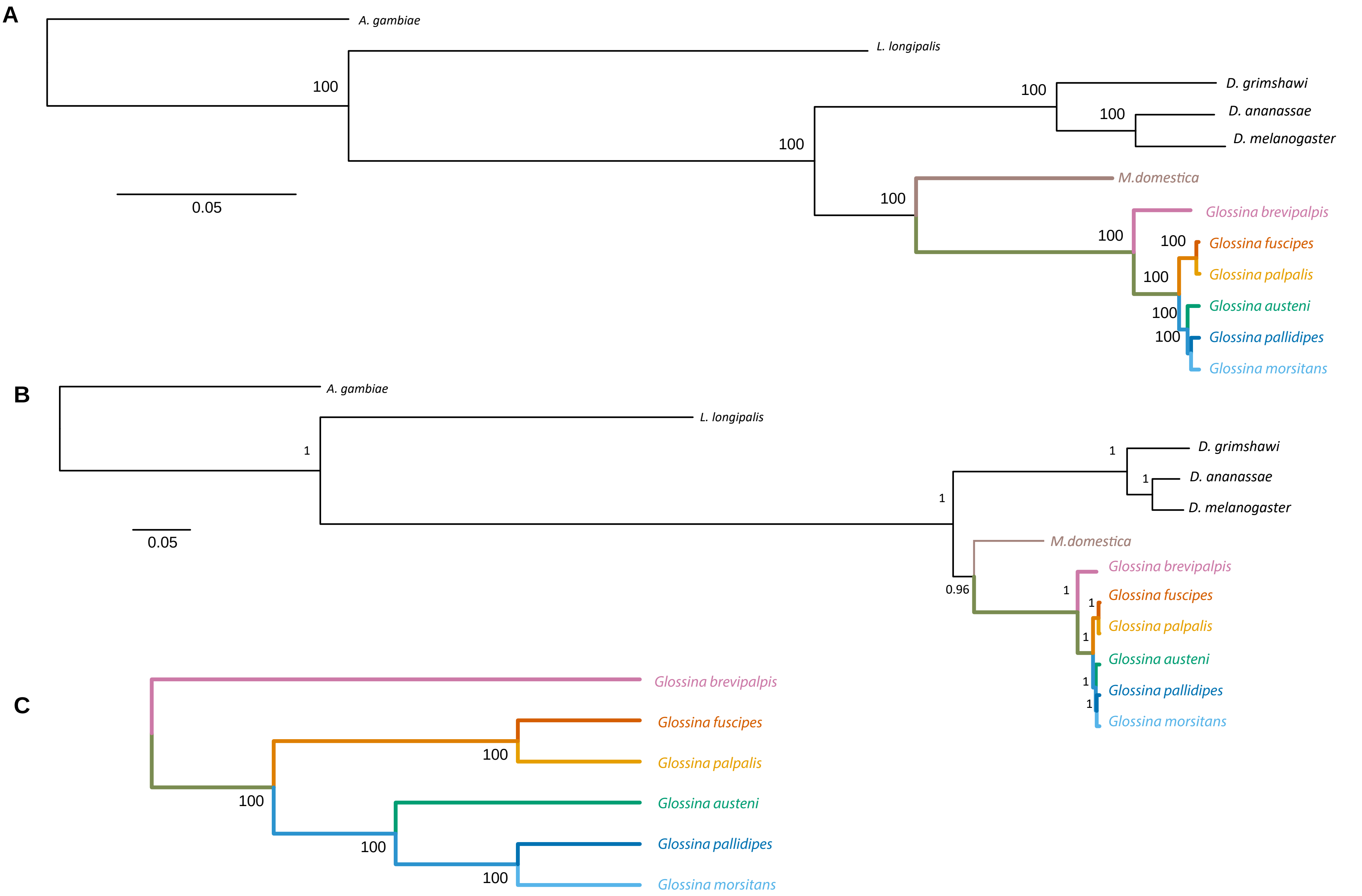

Suppl. Figure 1. A: Maximum likelihood tree inferred by Raxml on a concatenated alignment of 117,783 amino acids from 286 genes and employing the LG+G+F model of replacement. Value at nodes are bootstrap supports from the consensus of 100 pseudoreplicates in Raxml. B: Bayesian phylogentic tree inferred with Phylobayes 3 using CAT+G replacment model, supports at nodes are posterior probabilities; C: Astral analysis based on 286 genes with bootstrap support.

Supplemental Figure 3

A

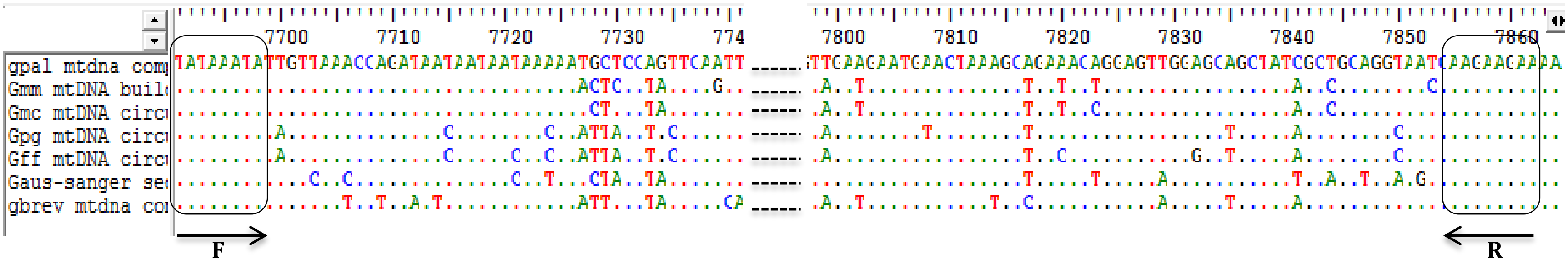

B

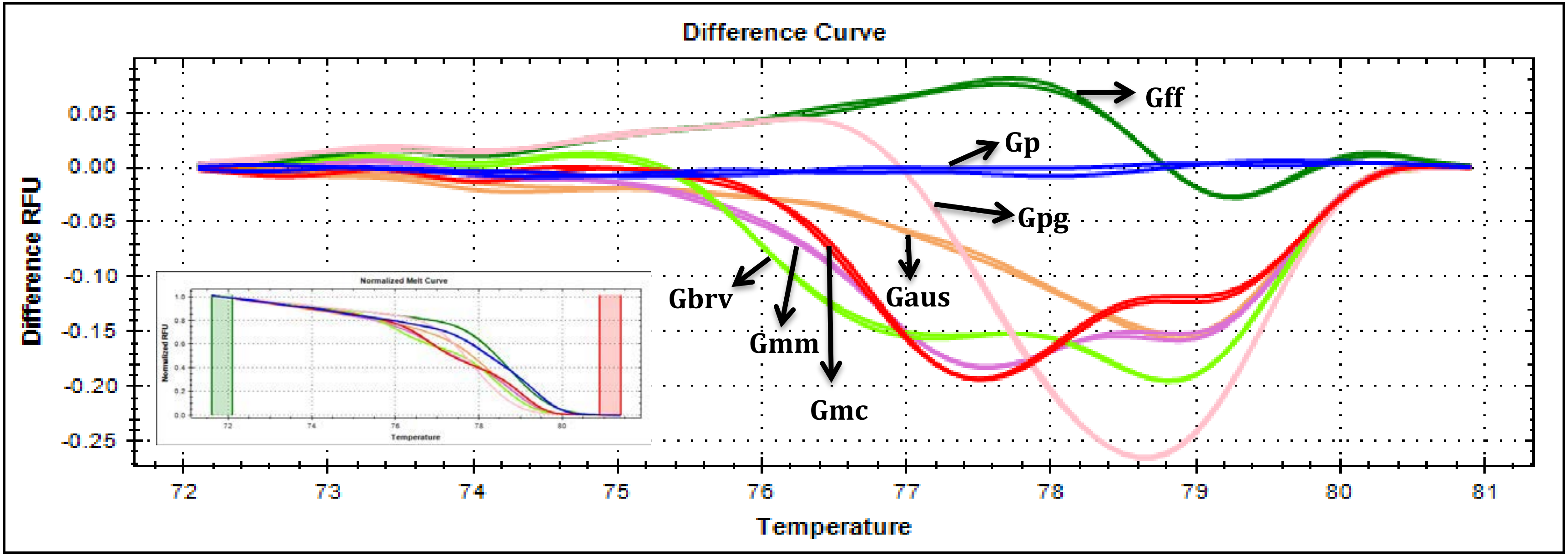

Supplemental Figure 4

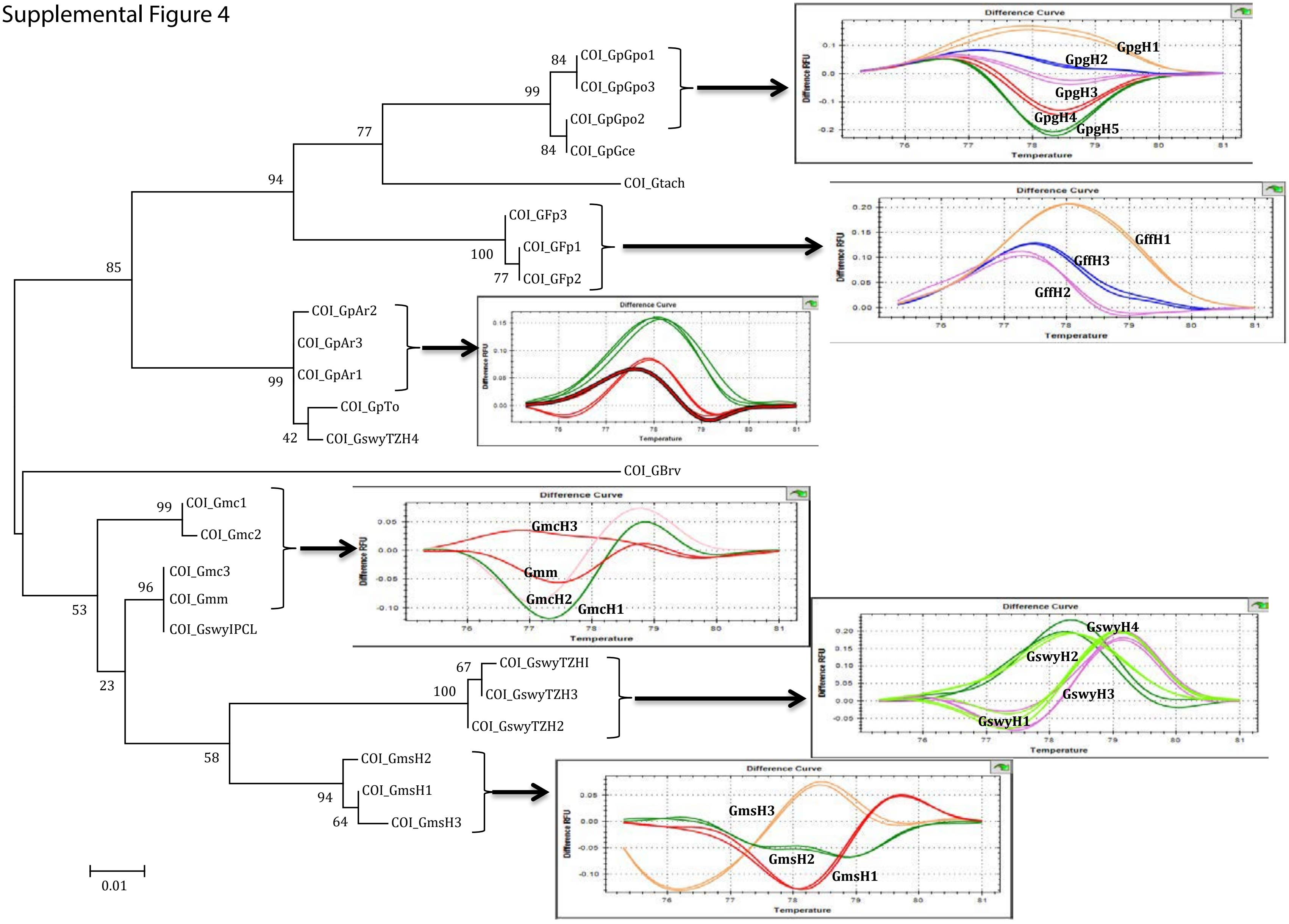

Supplemental Figure 5

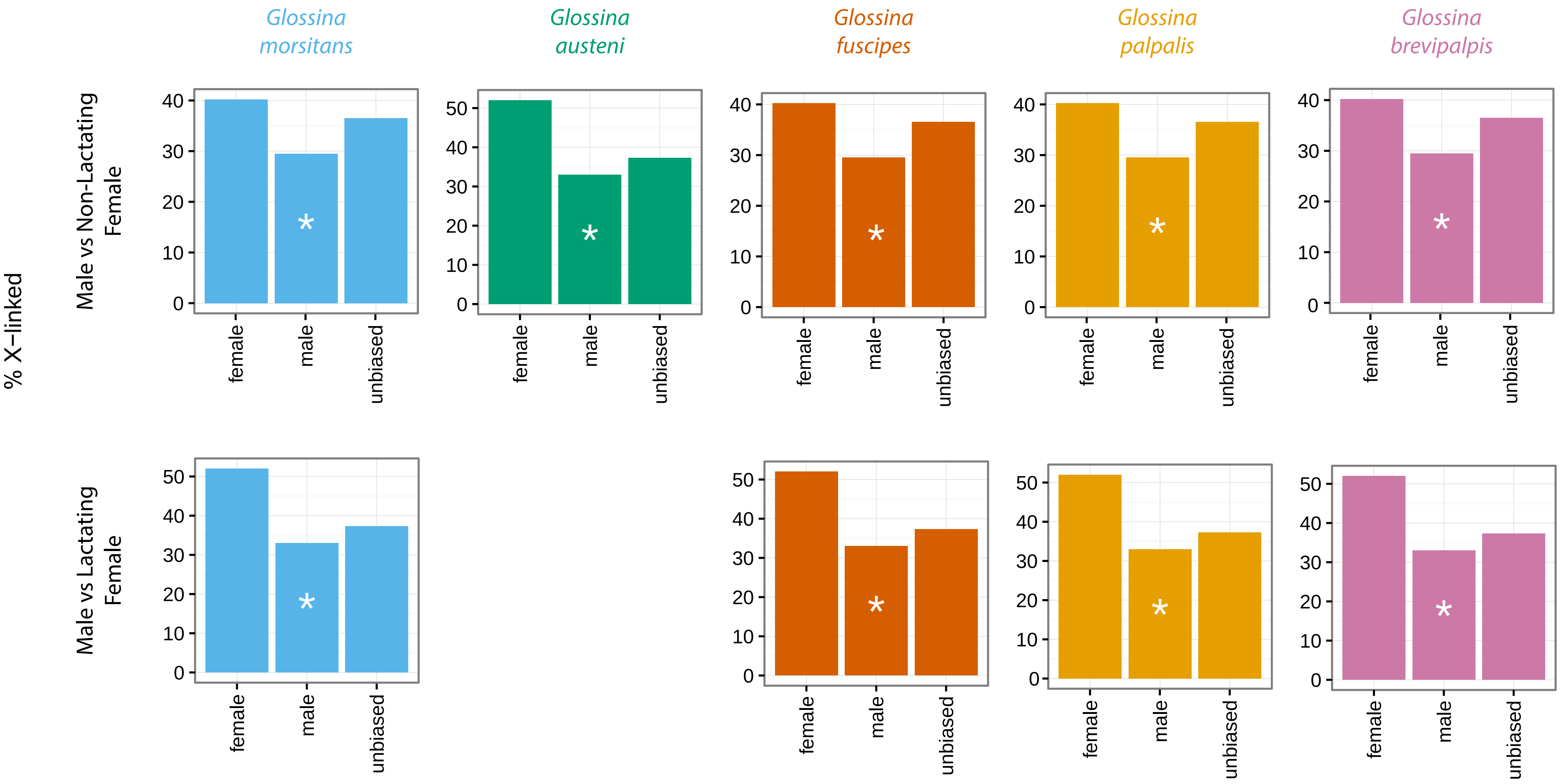

Supplemental Figure 6

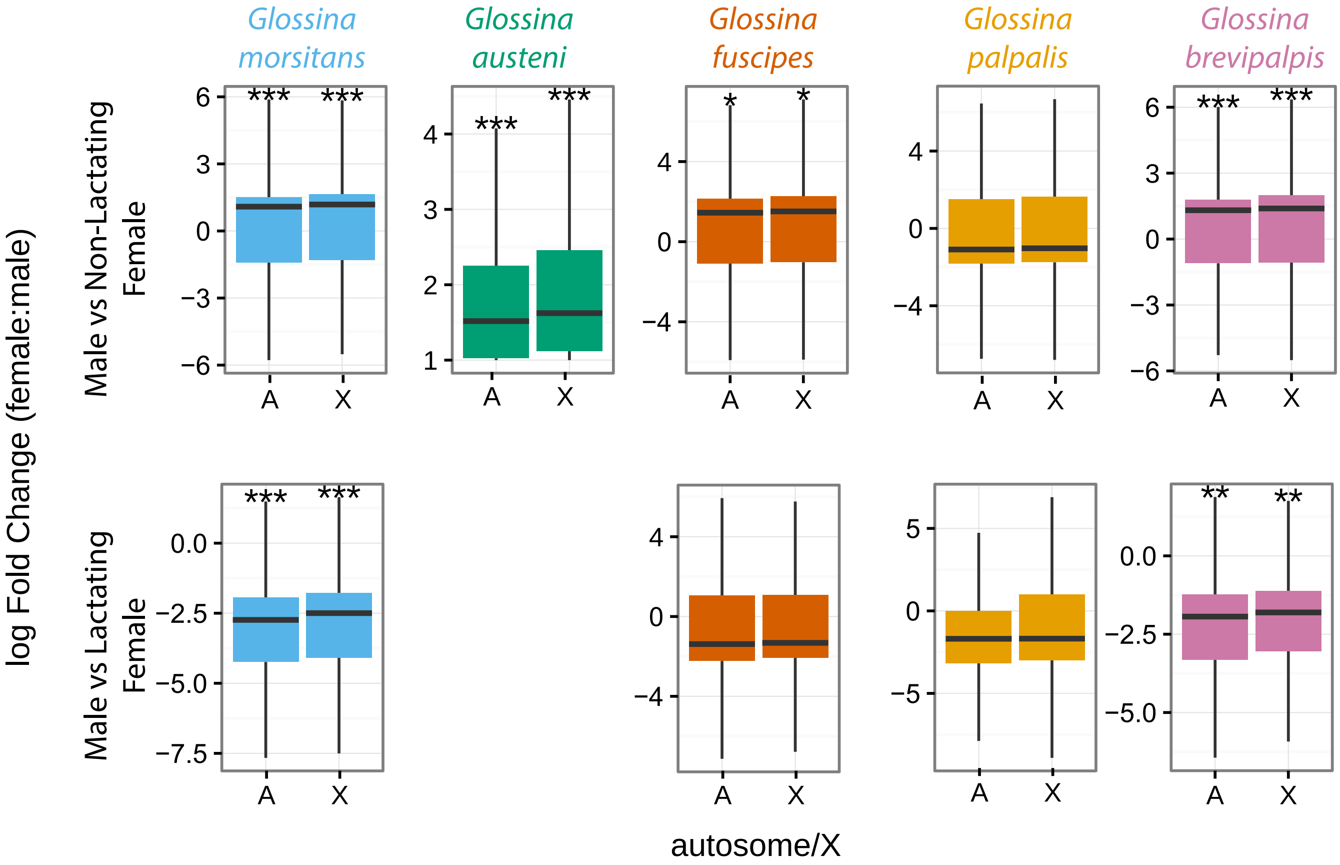

Supplemental Figure 7

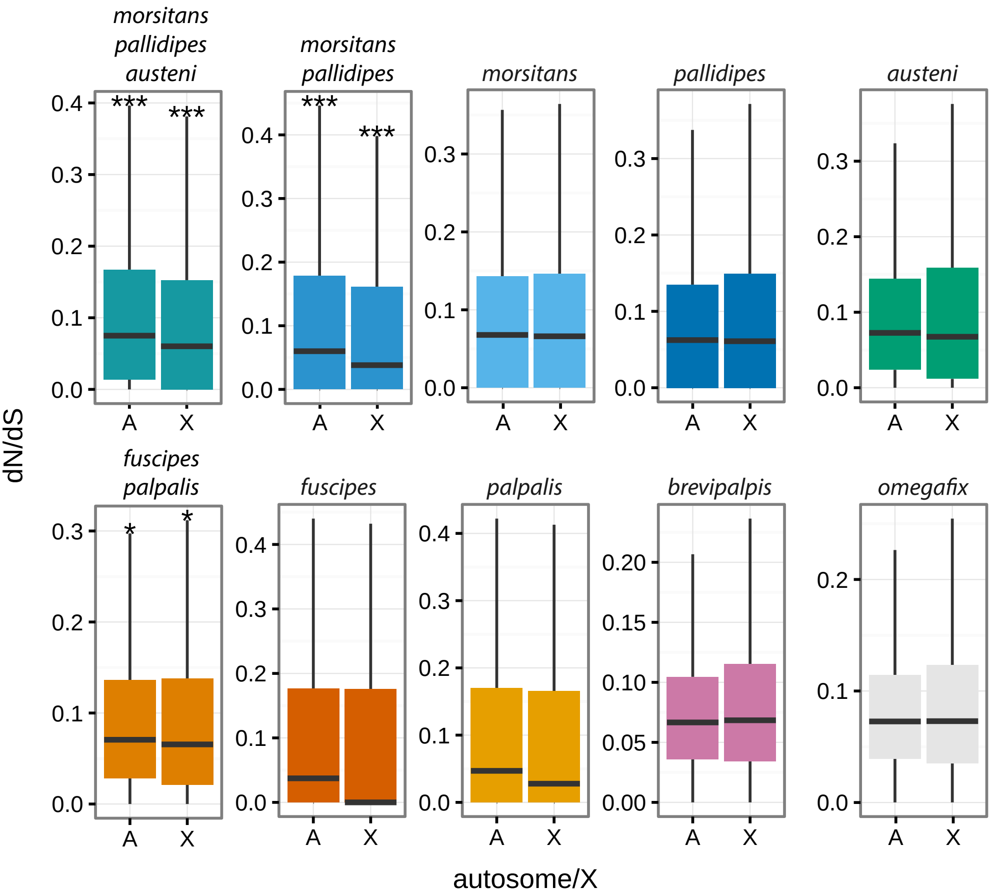

Supplemental Figure 8

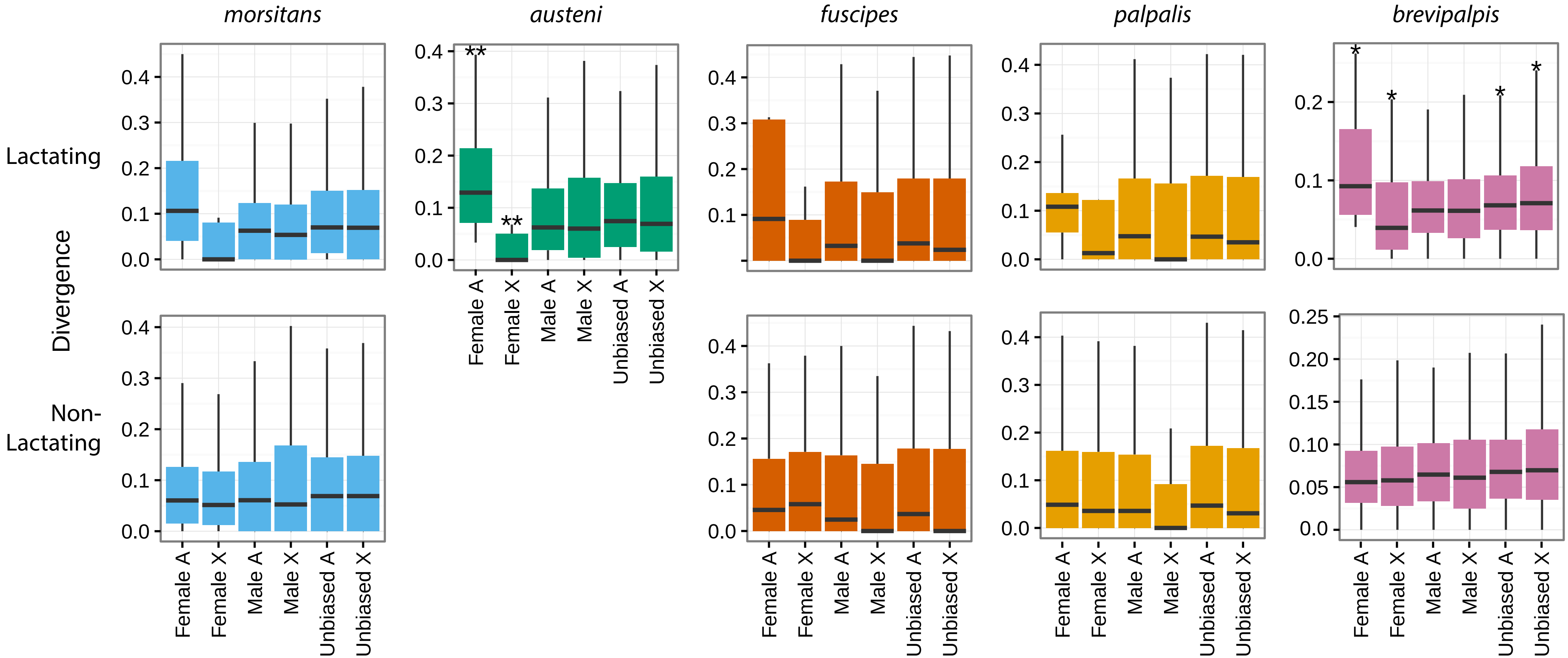

Supplemental Figure 9

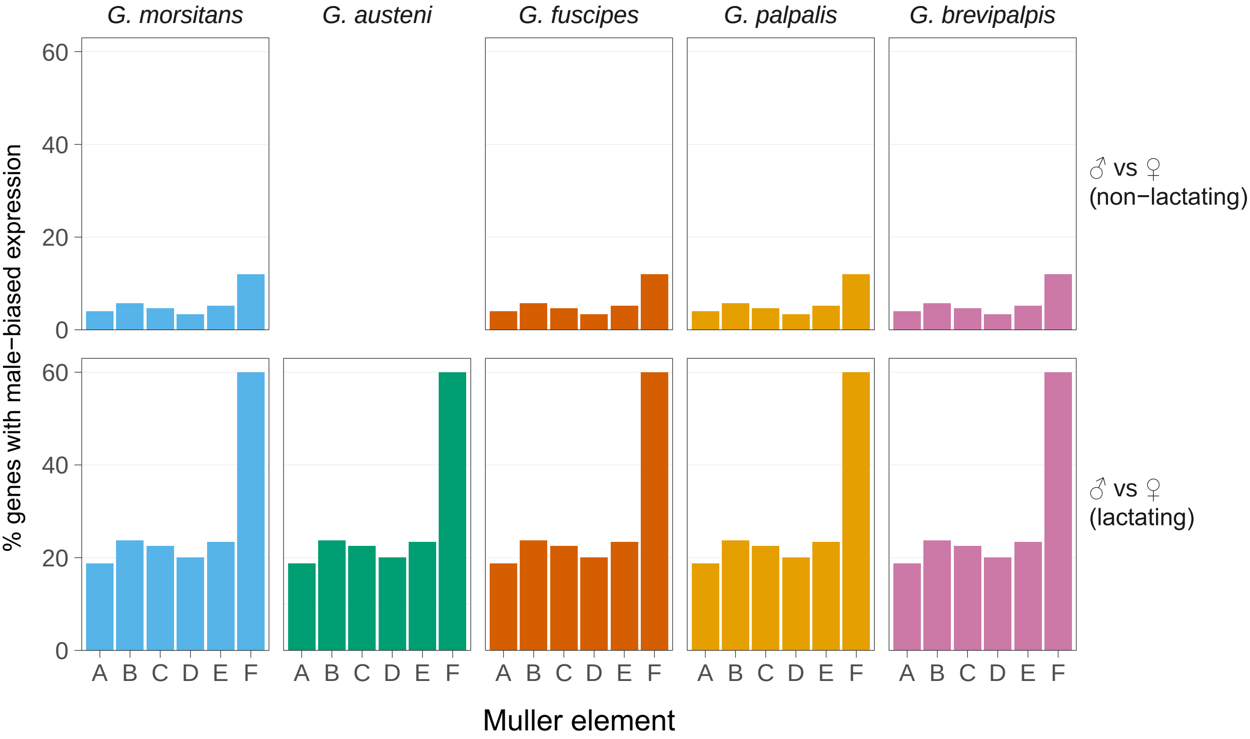

Supplemental Figure 10

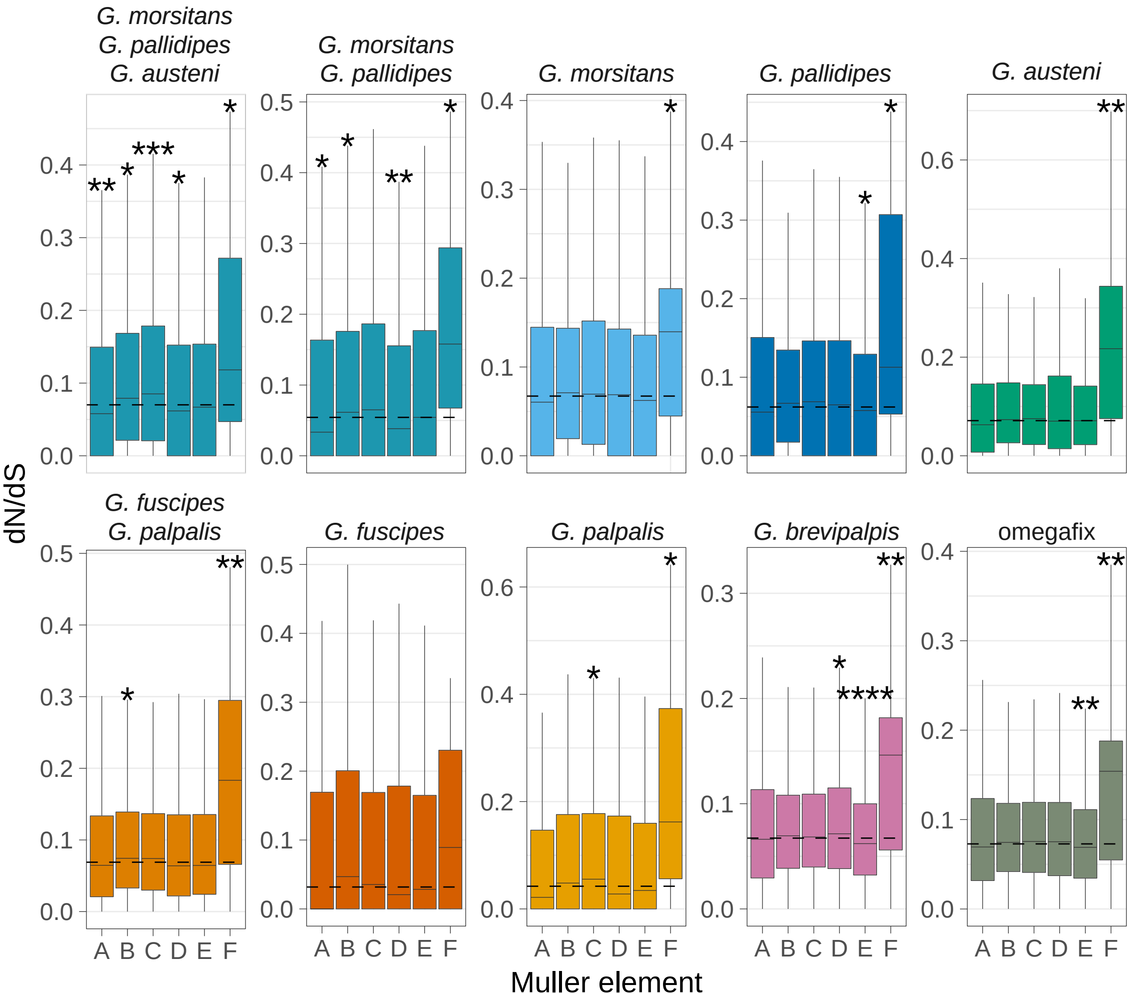

Supplemental Figure 11

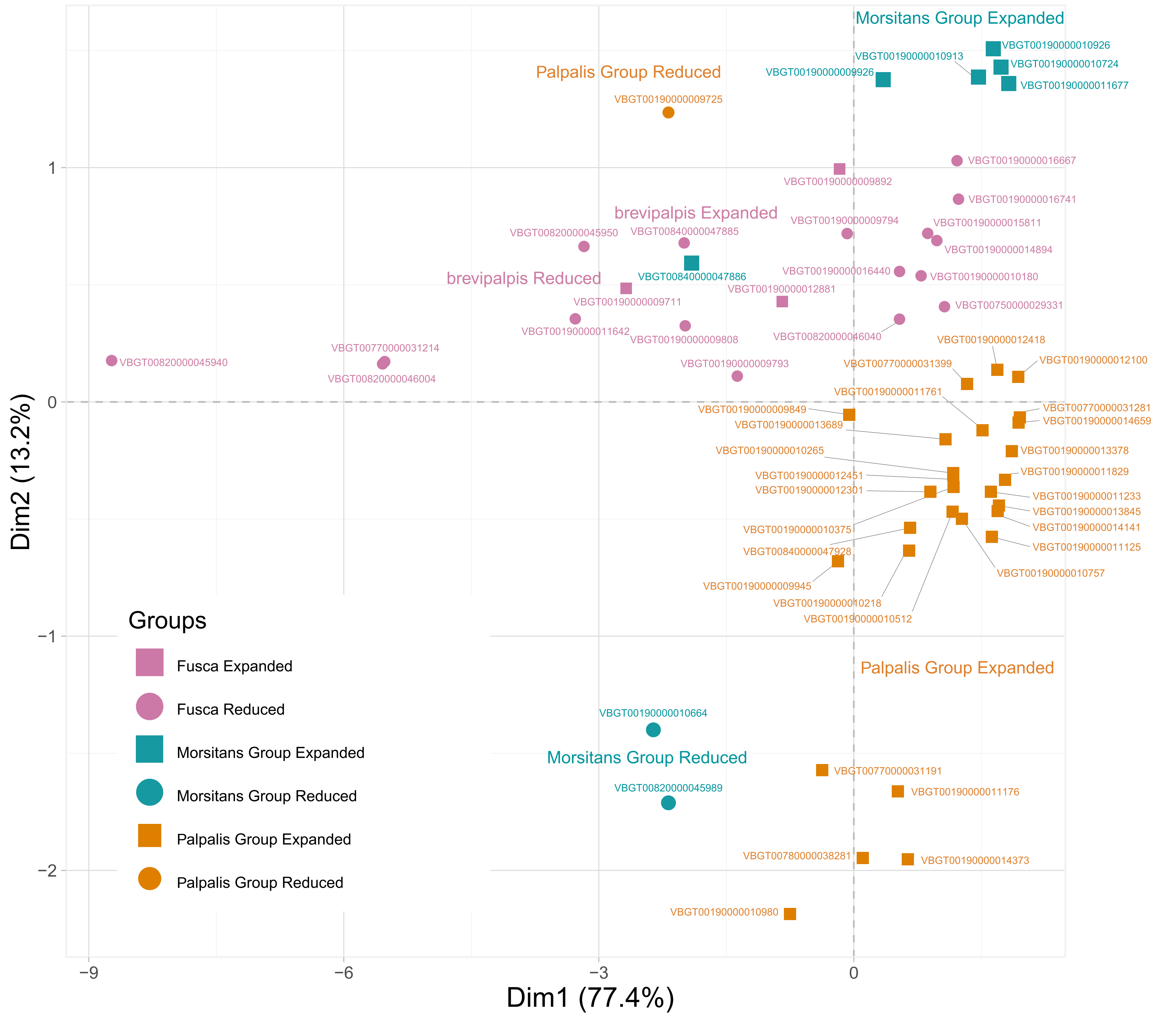

Supplemental Figure 12

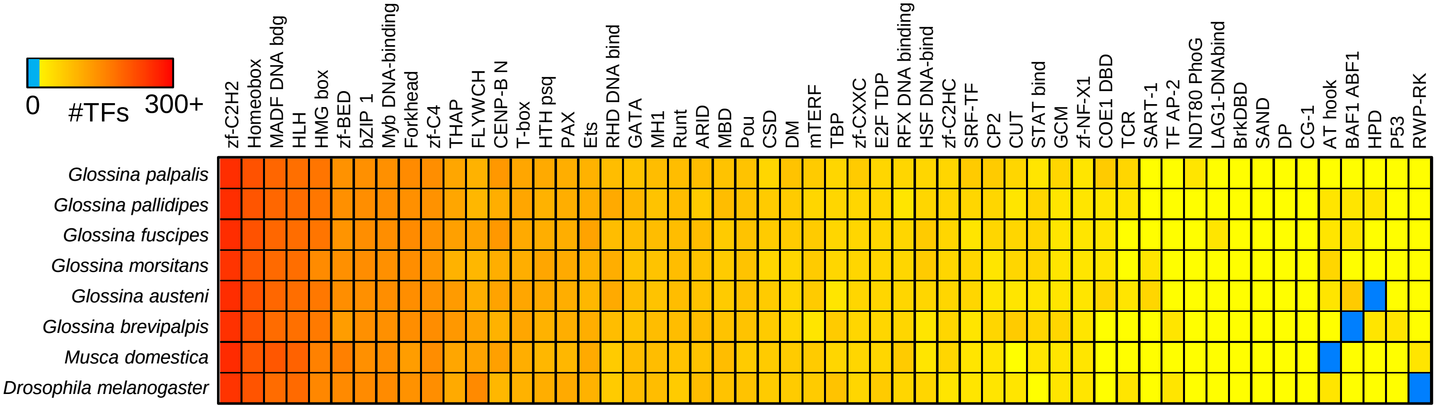

Supplement: Supplementary file 2 — Supplemental Figures S1-S12 and associated captions. (PDF 2260 kb) [file 13059_2019_1768_MOESM2_ESM.pdf]
