# Supplementary material for: Comparative genomic analysis of six Glossina genomes, vectors of African trypanosomes
Source: Genome Biol. 2019 Sep 2;20:187. doi: 10.1186/s13059-019-1768-2 (PMC6721284; doi:10.1186/s13059-019-1768-2)
Supplement: Supplementary file 13 — Review history. (DOCX 31 kb) [file 13059_2019_1768_MOESM13_ESM.docx]

**Review history**

**Reviewer 1**

Reviewer comments to the authors:

Authors have assembled 5 new Glossina genomes and combined them with the original Glossina morsitans genome, allowing for a comparative genomics study into a relatively understudied disease vector of great economic and public health importance. The study is a thorough and exhaustive comparison of the genomes of this clade, it makes good connections to previous work, the text is clear, and the conclusions are well founded. The work should be considered for publication in Genome Biology.

Specific points to be addressed: 

I have remaining questions over the completeness of these genome assemblies. I was unable to find a table for easy comparison of assembly / contig N50s, gap numbers, estimated repeat content - as the assembly of these genomes is at the core of this work, this table must be included (BUSCO completeness could also be included here). Similarly, there are methods (most of all Hi-C, but also bionano, 10X, potentially pacbio/nanopore) that have recently scaffolded challenging dipteran genomes end-to-end. Financial considerations might have discounted these, but it would be helpful to know if any were attempted or, if not, why not. 

Somewhat related, TE results (supplemental file 1) are likely very susceptible to genome completeness. Some comparison of repeat content and/or uniqueness of contigs would help in trusting the result here. In addition, is there evidence of any significant difference in TE Content between species? 

The section on expansion of immune families is well constructed, but this seems a good opportunity to draw some conclusions on the evolutionary constraints on the immune response. Are there any differences in dNdS between groups that might suggest contrasting responses? Or are there differences in evol rate for effector / pathogen recognition / signal transduction proteins? 

The paper is somewhat long. While core analyses such as the evolution of sex chromosomes, the expansions of proteases / immune families, and the evolution of milk proteins and rhodopsins are interesting, I feel that moving some of the sections into supplemental would aid the more casual reader.


Minor points:

L135 - colonial period may not be the most clear or useful time span to use; past 200 years or similar would be more appropriate. 

L178-9 - reference for sister group is missing or not obvious

L182 - guessing this should be "anthropophily"

L191 - sp 'experiments'

L194-198 - somewhat wordy retelling of assembly history, might be better to just ref genome paper?

L228-234 - low gene content of Brevipalpis is interesting if it results from loss, but less so if it results from poor assembly; it would be helpful to have some details on the assembled genome size / repeat content / BUSCO completeness here.

L235-238 - though it is available in the supplemental, no bootstrapping / posterior probs or other support is shown on this tree. This feels important enough to make clear to the reader what constituted 'support from ML / Bayesian analysis' here without recourse to supplemental

L242 - were any tests for introgression performed between clades? And if so how does this relate to any differences in topology?

L262-270 - again tests for introgression / abbababa etc seem to be an omission here. 

L308-310 - no evidence of faster-X, but this may not be expected if Muller element D has recently migrated to the x chromosome. Does lineage sorting between these clades allow for independent analysis of this? Or can the Autosomal dNdS be seen to be different for Muller element A?

L335-336 please rewrite for clarity

L364-365 please rewrite for clarity

L389-392 - this is a very interesting result, but given the bloodmeal microbiome, association with hematophagy is not incompatible with roles in immunity. Do any of the expanded families show homology to proteolysis genes identified by zhang, et al, or previously assigned to known immune pathways? 

Figures:

Fig2 b/c - bootstrap /posterior prob values would be helpful here, rather than in supp. (see also comment on lines 235-238). 

Fig4 - circos plot is highly fragmented, and difficult to see any meaningful pattern (unless the fragmentation is the meaningful pattern); potentially due to degradation of the inserted sequence? Would larger blocks at lower pc identity show this more clearly? 

Fig 6 - I'm honestly not sure what this PCA is attempting to convey; I think a table of expanded/contracted families would convey this information more usefully

Fig 7 - clearer descriptions of how these separate heatmaps were derived would be useful. Whether any of these expansions relate to specific immune pathways / or classes of pathogen-recognition or effector proteins would be useful. 

Fig 8c - mean fold change is a bit of a blunt measure; error bars would at least help with interpretation of this result. 

Fig 9 - needs separate descriptions of Fig a/b in the legend as well as of the significance measure in Fig 9a. Fig 9b would be far more useful with functional classes described (where known). 


Supplemental file 1:
Repeat analysis, Para4, line 1-2: "helitrons assoc with…" should be referenced.

**Reviewer 2**

This manuscript by Attardo et al. describes the comparative genomic analysis of 6 Glossina species genomes in 3 subgenera. Glossina display multiple unique adaptations or phenotypes, including live-birthing, male and female hematophagy, obligate bacterial symbionts, and transmission of trypanosomes.

Five new Glossina genome assemblies were generated in the study. The assemblies were annotated and analyzed with respect to each other, Musca, and Drosophila. The analysis is highly descriptive, as appropriate for the goals of the study, but there are a number of interesting biological hypotheses presented that would be worthy of followup. The work also yielded a proposed new method for tsetse diagnostics using HRM analysis of mtDNA. 
By the author list, there is a good balance of biological expertise as well as bioinformatic firepower. Both are evident in the ms, which is well grounded in the biology of Glossina ecology and pathogen transmission, and is also expertly analyzed in the phylogenomics. 

The work represents an important contribution, and can be published with a few minor corrections. I do not consider that this rises to the level that requires a formal revision. The analysis of immunity genes is interesting and highlights loss and expansion of immune factors. The finding of polydnavirus-related sequences in G brevipalpis, combined with the possible enhancement of genes involved in encapsulation, points to a potentially interesting ecological exposure of this species to parasitoid wasps. 

I have some minor points for correction:
line 335: "Furthermore, analysis of fly lines from which the sequenced DNA was obtained with Wolbachia specific primers PCR-amplification was negative." This sentence should be reworded as it is not really clear what it means. The rest of this paragraph and the next para also needs work to clarify what they are trying to say about genomic sequences related to Wolbachia. Are they evolutionary stable components of the Glossina genome, are they expressed, do they behave like mobile elements, or are they punctual, accidental HGT events resulting from the intimate symbiosis, but otherwise lacking biological meaning?

line 386: "lowest p-value" not safe to say because not clear if it means "most significant p-value" or the opposite, I think they mean the former but please correct. 

line 543: polydnavirus typo was written as polynavirus

**Response to reviewers**

**Reviewer #1**

*Authors have assembled 5 new Glossina genomes and combined them with the*

*original Glossina morsitans genome, allowing for a comparative genomics study into a*

*relatively understudied disease vector of great economic and public health importance.*

*The study is a thorough and exhaustive comparison of the genomes of this clade, it*

*makes good connections to previous work, the text is clear, and the conclusions are*

*well founded. The work should be considered for publication in Genome Biology.*

*Specific points to be addressed:*

*I have remaining questions over the completeness of these genome assemblies. I was*

*unable to find a table for easy comparison of assembly / contig N50s, gap numbers,*

*estimated repeat content - as the assembly of these genomes is at the core of this*

*work, this table must be included (BUSCO completeness could also be included here).*

*Similarly, there are methods (most of all Hi-C, but also bionano, 10X, potentially*

*pacbio/nanopore) that have recently scaffolded challenging dipteran genomes end-toend.*

*Financial considerations might have discounted these, but it would be helpful to*

*know if any were attempted or, if not, why not.*

*Somewhat related, TE results (supplemental file 1) are likely very susceptible to*

*genome completeness. Some comparison of repeat content and/or uniqueness of*

*contigs would help in trusting the result here. In addition, is there evidence of any*

*significant difference in TE Content between species?*

•We apologize for not having including this information in the main manuscript. The

write up on the assembly parameters, the BUSCO analyses, the repeat analyses and

the associated data tables were in the Supplemental Materials. We have moved this

information back into the main body of the text to provide context on the assemblies

which is summarized in Figure 2 which has also been moved into the main text and

improved with an additional panel displaying the proportion of shared TE families.

Detailed tables including comparative statistics the different classes of repetitive

elements have been generated and included within Additional file 1 –Supplemental

Tables 3+4

•In regards to the use of more modern long read technologies for improving these

assemblies, at the time when the sequencing for this project was being performed

many of the suggested technologies were in either in very early stages or did not yet

exist. Unfortunately, all the genetic material that was collected from each species was

utilized in the original sequencing effort. Current funds for additional sequencing and

assembly improvement efforts are limited. However, we are aiming to improve the assemblies for these genomes in the future and work is in progress for the

improvement of individual species.

*The section on expansion of immune families is well constructed, but this seems a*

*good opportunity to draw some conclusions on the evolutionary constraints on the*

*immune response. Are there any differences in dNdS between groups that might*

*suggest contrasting responses? Or are there differences in evol rate for effector /*

*pathogen recognition / signal transduction proteins?*

•We have added the average dN/dS ratios for the immune gene families where

available. Some were not able to be calculated due to poor alignment quality. This

could be the result of significant sequence variability or low quality gene annotations

which will require additional refinements. However, we did identify significant

differences in variation in genes associated with immune system repression. We have

modified the immunity section to reflect this and to describe the function of these

genes.

*The paper is somewhat long. While core analyses such as the evolution of sex*

*chromosomes, the expansions of proteases / immune families, and the evolution of*

*milk proteins and rhodopsins are interesting, I feel that moving some of the sections*

*into supplemental would aid the more casual reader.*

•At the request of the editor we have left these sections in the manuscript.

*Minor points:*

*L135 - colonial period may not be the most clear or useful time span to use; past 200*

*years or similar would be more appropriate.*

•We have updated this statement to be more specific/accurate in terms of when record

keeping regarding Trypanosomiasis cases began (Lines 135-137).

*L178-9 - reference for sister group is missing or not obvious*

•We have added in a reference defining the previous phylogenetic characterization of

the Fusca sub-genus as a sister group by sequence analyses of 2 mitochondrial

(cytochrome oxidase 1, 16S ribosomal RNA) and 2 nuclear genetic markers (NADH

dehydrogenase subunit 2 (ND2) and the Internal transcribed spacer 1 of ribosomal

DNA (ITS1)).

*L182 - guessing this should be "anthropophily"*

•Corrected

*L191 - sp 'experiments'*

•Corrected

*L194-198 - somewhat wordy retelling of assembly history, might be better to just ref*

*genome paper?*

•This section has been reduced to eliminate redundant or unnecessary details

available in the original genome paper. The description of the species used has also

been revised to clarify the justification for their selection (Lines 197-200).

*L228-234 - low gene content of Brevipalpis is interesting if it results from loss, but less*

*so if it results from poor assembly; it would be helpful to have some details on the*

*assembled genome size / repeat content / BUSCO completeness here.*

•We have included the assembly statistics and associated BUSCO analyses requested

in comments above to the main text to provide additional context to aid in interpretation

of this section. Based on the N50 and L50 values all the assemblies appear to be of

high quality. In particular, the G. brevipalpis assembly ranks 2nd out of the six in terms

of those scores. The BUSCO scores for the G. brevipalpis assembly also indicate the

assembly in it current form represents a comprehensive representation of the genome

with only 1.89% and 0.79% of BUSCOs missing from the predicted gene set and whole

genome respectively.

*L235-238 - though it is available in the supplemental, no bootstrapping / posterior*

*probs or other support is shown on this tree. This feels important enough to make clear*

*to the reader what constituted 'support from ML / Bayesian analysis' here without*

*recourse to supplemental*

•We have updated Figure 2B to include the representative bootstrap values.

*L242 - were any tests for introgression performed between clades? And if so how does*

*this relate to any differences in topology?*

*L262-270 - again tests for introgression / abbababa etc seem to be an omission here.*

•We tested for introgression using Astral (figure 2B, and suppl figure 1C) which

compares single gene typologies. The analysis reveals perfect (maximal bootstrap

support) congruence among the many genes indicating no signs of past recombination

events (ie: complete sorting for these markers). This is reported in lines 307-309 in the

current version. For more clarity we have slightly modified the text in this section to: "A

coalescent-aware analysis further returned full support, indicating a speciation process

characterized by clear lineage sorting with no introgression between species

(Supplemental Figure 1).”

*L308-310 - no evidence of faster-X, but this may not be expected if Muller element D*

*has recently migrated to the x chromosome. Does lineage sorting between these*

*clades allow for independent analysis of this? Or can the Autosomal dNdS be seen to*

*be different for Muller element A?*

•Almost all Glossina spp. in our study have the same karyotype: two large autosomal

pairs (L1 and L2) and a large X chromosome (Maudlin 1970; Southern & Pell 1974;

Willhoeft 1997). There are some species (or populations within species) that have

supernumerary chromosomes (Southern & Pell 1974; Maudlin 1979), however, the

primary focus of this paper is on the three main chromosomes. The one key exception

to the standard karyotype in the species we have sampled is G. brevipalpis (our

outgroup), which has 5 large chromosomes with substantial euchromatic regions and

2-12 smaller heterochromatic elements (Maudlin 1970; Willhoeft 1997). The G.

brevipalpis karyotype is similar to the ancestral karyotype of brachyceran flies (higher

diptera), which consists of five large, euchromatic elements and a heterochromatic sex

chromosome pair (Vicoso & Bachtrog 2013). The heterochromatic elements in G.

brevipalpis could correspond to sex chromosomes and/or supernumerary

chromosomes.

The X chromosome in G. morsitans consists of Muller elements A, D, and F (Vicoso &

Bachtrog 2015). Element F is the ancestral X, and elements A and D are recent

additions to the X chromosome that happened sometime after the split between

Glossina and the rest of the calyptrates (Vicoso & Bachtrog 2013). However, in

Drosophila element A has become X-linked independently of other calyptrate flies. The

fact that all Glossina spp. other than G. brevipalpis share the same karyotype suggests

that the same elements are X-linked in all species other than G. brevipalpis. It’s

parsimonious to assume that element F is X-linked in G. brevipalpis because it is Xlinked

in the other Glossina spp., in closely related calyptrates, and in more distantly

related flies (including the inferred common ancestor). However, we do not know which

other elements are X-linked in G. brevipalpis. So, any odd results in G. brevipalpis

could be explained by our uncertainty about what is X-linked in that species. However,

we can be fairly confident about our inference of X-linked elements in the other

species.

References:

Maudlin I (1970). Preliminary studies on the karyotypes of five species of Glossina.

Parasitology 61: 71-74

Maudlin I (1979). Chromosome polymorphism and sex determination in a wild

population of tsetse. Nature 277: 300-301

Southern DI, Pell PE (1974). Comparative Analysis of the Polytene Chromosomes of

Glossina austeni and Glossina morsitans morsitans. Chromosome 47: 213-226

Vicoso B, Bachtrog D (2013). Reversal of an ancient sex chromosome to an autosome

in Drosophila. Nature 499: 332-335

Vicoso B, Bachtrog D (2015). Numerous transitions of sex chromosomes in Diptera.

PLOS Biol 13: e1002078

Willhoeft U (1997). Fluorescence in situ hybridization of ribosomal DNA to mitotic

chromosomes of tsetse flies (Diptera: Glossinidae: Glossina). Chromosome Res 5:

262-267

*L335-336 please rewrite for clarity*

•This section has been rewritten for clarity.

*L364-365 please rewrite for clarity*

•This section has been rewritten for clarity.

*L389-392 - this is a very interesting result, but given the bloodmeal microbiome,*

*association with hematophagy is not incompatible with roles in immunity. Do any of the*

*expanded families show homology to proteolysis genes identified by zhang, et al, or*

*previously assigned to known immune pathways?*

•We have not made direct comparisons between the protease orthologs in tsetse

relative to those identified in the Zhang paper to determine if these sequences are

ortholous or if the expansions in Glossina were derived from an independent set of

genes. BLAST analysis of these sequences suggests that most of these sequences

are most homologus to uncharacterized chymotrypsins and trypsins in other

Brachyceran Diptera. We felt that an in depth functional analysis of these sequences

would be beyond the scope of this paper. However, this will be something that we

investigate in future work to determine the evolutionary origins of these proteins, as

well as their tissue and stage specificities to identify their putative functions. We have

added some additional material to the manuscript describing the results of the

homology analysis and discuss of the potential functions these genes might be

associated with (lines 485-491).

*Figures:*

*Fig2 b/c - bootstrap /posterior prob values would be helpful here, rather than in supp.*

*(see also comment on lines 235-238).*

•Figure 2 and its caption have been updated to reflect the bootstrap and posterior

probability values derived from the maximum likelihood and bayesian phylogenetic

analyses.

*Fig4 - circos plot is highly fragmented, and difficult to see any meaningful pattern*

*(unless the fragmentation is the meaningful pattern); potentially due to degradation of*

*the inserted sequence? Would larger blocks at lower pc identity show this more*

*clearly?*

•The function of this plot is meant to highlight regions of the Glossina genomes that

have maintained their genetic structure (synteny) as well as regions that have

undergone significant restructuring relative to Drosophila melanogaster. The figure

has been replotted using a 250 kB block size to attempt to consolidate the data and

make the figure more easily interpretable.

*Fig 6 - I'm honestly not sure what this PCA is attempting to convey; I think a table of*

*expanded/contracted families would convey this information more usefully*

•This information was rendered graphically as a way to visualize the amount of

variation within and between the expanded and contracted orthology groups. We have

updated the figure to a version which contains identifying information for each point

which should be more informative. An equivalent plot with the orthology group IDs is

also included as a supplemental figure. The table containing the raw data from which

these plots are derived has also been included in Additional File 1 – Supplemental

Table 4. We felt that this table was probably too long to include in the body of the main

text.

*Fig 7 - clearer descriptions of how these separate heatmaps were derived would be*

*useful. Whether any of these expansions relate to specific immune pathways / or*

*classes of pathogen-recognition or effector proteins would be useful.*

•This figure represents immune gene families that display a variance greater than 1 in

the number of orthologs/paralogs between species. The immune gene heatmaps were

created using the R package pHeatmap. This package creates a dendrogram based

on the similarity of the features as determined by Pearson correlation. The 4 heatmaps

represent gene orthology families with similar patterns of ortholog numbers per

species. These patterns indicate species or sub-genus specific gene expansions or

contractions. In the case of this figure the four panels represent gene families with

expansions in Musca domestica, the Palpalis sub-genus, Glossina austeni and

Glossina brevipalpis respectively. Lines 572-576 have been added to clarify the

derivation of the figure and the clustering. The figure caption has also been updated to

clarify its content and derivation.

*Fig 8c - mean fold change is a bit of a blunt measure; error bars would at least help*

*with interpretation of this result.*

•Unfortunately, we only had the resources to generate one replicate of each

reproductive state per species, so we were not able to perform stats and can only

make direct comparisons between the two states in terms of fold difference.

*Fig 9 - needs separate descriptions of Fig a/b in the legend as well as of the*

*significance measure in Fig 9a. Fig 9b would be far more useful with functional classes*

*described (where known).*

•The legend for figure 9 has been rewritten to describe both parts of the figure. The

error bars a part A represent standard error and the asterisks represent a p- value of <

10e-5 as determined by the Wilcoxon Rank-Sum test. This information has been added

to the figure legend. The section for part B has been updated with descriptions of the

functional class abbreviations used in the figure.

*Supplemental file 1:*

*Repeat analysis, Para4, line 1-2: "helitrons assoc with…" should be referenced.*

•We have updated the text with the appropriate reference which describes the

association of heliotron activity with vertical transmission in Drosophila.

**Reviewer #2:**

*This manuscript by Attardo et al. describes the comparative genomic*

*analysis of 6 Glossina species genomes in 3 subgenera. Glossina display multiple*

*unique adaptations or phenotypes, including live-birthing, male and female*

*hematophagy, obligate bacterial symbionts, and transmission of trypanosomes.*

*Five new Glossina genome assemblies were generated in the study. The assemblies*

*were annotated and analyzed with respect to each other, Musca, and Drosophila. The*

*analysis is highly descriptive, as appropriate for the goals of the study, but there are a*

*number of interesting biological hypotheses presented that would be worthy of*

*followup. The work also yielded a proposed new method for tsetse diagnostics using*

*HRM analysis of mtDNA.*

*By the author list, there is a good balance of biological expertise as well as*

*bioinformatic firepower. Both are evident in the ms, which is well grounded in the*

*biology of Glossina ecology and pathogen transmission, and is also expertly analyzed*

*in the phylogenomics.*

*The work represents an important contribution, and can be published with a few minor*

*corrections. I do not consider that this rises to the level that requires a formal revision.*

*The analysis of immunity genes is interesting and highlights loss and expansion of*

*immune factors. The finding of polydnavirus-related sequences in G brevipalpis,*

*combined with the possible enhancement of genes involved in encapsulation, points to*

*a potentially interesting ecological exposure of this species to parasitoid wasps.*

*I have some minor points for correction:*

*line 335: "Furthermore, analysis of fly lines from which the sequenced DNA was*

*obtained with Wolbachia specific primers PCR-amplification was negative." This*

*sentence should be reworded as it is not really clear what it means. The rest of this*

*paragraph and the next para also needs work to clarify what they are trying to say*

*about genomic sequences related to Wolbachia. Are they evolutionary stable*

*components of the Glossina genome, are they expressed, do they behave like mobile*

*elements, or are they punctual, accidental HGT events resulting from the intimate*

*symbiosis, but otherwise lacking biological meaning?*

•This region has been reworded for clarity. We have also added in additional text (lines

425-430) which references and describes previous analyses relative to the biological

significance of the Wolbachia insertions identified in G. morsitans.

*line 386: "lowest p-value" not safe to say because not clear if it means "most significant*

*p-value" or the opposite, I think they mean the former but please correct.*

•This sentence has been reworded to clarify that the ontology categories being

described represent those with the most significant p-values (Lines 465-467)

*line 543: polydnavirus typo was written as polynavirus*

•Corrected
